# Can live microbial interventions support healthy aging? A systematic review and meta-analysis of cognitive and muscle outcomes in aging adults

**DOI:** 10.3389/fnut.2026.1869929

**Published:** 2026-09-16

**Authors:** Francisco Silva-Olivares, Nicole Vidal, Paula Acevedo-Hernández, Virginia Rabbia, Fanny Petermann-Rocha, Samanta Thomas-Valdés, Gonzalo Jorquera

**Affiliations:** 1 Instituto de Nutrición y Tecnología de los Alimentos (INTA), Universidad de Chile, Santiago, Chile; 2 Facultad de Salud y Ciencias Sociales, Universidad de las Américas, Sede Providencia, Santiago, Chile; 3 Centro de Neurobiología y Fisiopatología Integrativa (CENFI), Instituto de Fisiología, Universidad de Valparaíso, Valparaíso, Chile; 4 Centro Interdisciplinario de Neurociencia de Valparaíso (CINV), Universidad de Valparaíso, Valparaíso, Chile; 5 Centro de Estudios Traslacionales en Estrés y Salud Mental (C-estrés), Universidad de Valparaíso, Valparaíso, Chile; 6 Farncombe Family Digestive Health Research Institute, McMaster University, Hamilton, ON, Canada; 7 Centro de Investigación Biomédica, Facultad de Medicina, Universidad Diego Portales, Santiago, Chile; 8 Centro de Micro-Bioinnovación, Escuela de Nutrición y Dietética, Facultad de Farmacia, Universidad de Valparaíso, Valparaíso, Chile; 9 Instituto de Fisiología, Universidad de Valparaíso, Valparaíso, Chile

**Keywords:** aging, cognition, muscle strength, probiotics, synbiotics

## Abstract

**Introduction:**

Preserving cognitive and physical function is central to healthy aging, and the gut microbiota may influence both brain and muscle physiology. We evaluated whether live microorganism-based interventions affect cognitive and muscle-related outcomes in aging adults.

**Methods:**

We systematically reviewed randomized placebo-controlled trials of probiotics, synbiotics, or fecal microbiota transplantation in populations with a mean age of at least 60 years. MEDLINE, Embase, Web of Science, and the Cochrane Library were searched. Random-effects meta-analyses used Hedges’ g when at least three studies or independent comparisons reported sufficiently comparable outcomes. The protocol was prospectively registered in PROSPERO (CRD420251065993; https://www.crd.york.ac.uk/PROSPERO/view/CRD420251065993).

**Results:**

Twenty-four trials involving 1,809 participants were included qualitatively: 20 evaluated cognitive outcomes and four evaluated muscle-related outcomes. Eleven unique studies contributed to at least one meta-analysis. For Mini-Mental State Examination scores, the overall pooled estimate was positive (*g* = 1.02; 95% CI: 0.36–1.68; *I*^2^ = 90.84%). In exploratory *post hoc* subgroup analyses, the estimate was positive in Alzheimer’s disease (*g* = 1.46; 95% CI: 1.14–1.79) but not statistically significant in broader cognitive impairment (*g* = 0.49; 95% CI: −0.78 to 1.77); the subgroup difference was not significant (p = 0.15). Excluding both Akhgarjand et al. intervention arms yielded a positive estimate in Alzheimer’s disease (*g* = 1.63; 95% CI: 1.21–2.06), although only two studies remained. The pooled Repeatable Battery for the Assessment of Neuropsychological Status result was not statistically significant (*g* = 0.46; 95% CI: −0.50 to 1.41). Handgrip strength showed a modest positive estimate (*g* = 0.46; 95% CI: 0.06–0.85), but exclusion of Qaisar et al. attenuated it to non-significance (*g* = 0.23; 95% CI: −0.27 to 0.73).

**Discussion:**

Live microorganism-based interventions showed preliminary short-term functional signals, but the small evidence base, high cognitive heterogeneity, short follow-up, and sensitivity to individual studies preclude firm efficacy conclusions. Subgroup and two-study sensitivity findings are exploratory. No eligible fecal microbiota transplantation trial was identified, so the evidence derives exclusively from probiotic and synbiotic studies.

**Systematic review registration:**

https://www.crd.york.ac.uk/PROSPERO/view/CRD420251065993, unique identifier: CRD420251065993.

## 1 Introduction

In the World Health Organization (WHO) framework, healthy aging is understood as the process of developing and maintaining the functional ability that enables wellbeing in older age, a concept that depends on both physical and mental capacities (1). From this perspective, maintaining cognitive and physical function is essential for sustaining independence, social participation, autonomy, and quality of life in older adults.

Among the biological systems increasingly implicated in healthy aging, the gut microbiota has emerged as a relevant modulator of host physiology. Aging is associated with alterations in gut microbial composition, intestinal barrier integrity, immune signaling, and microbial metabolite production, all of which may contribute to chronic low-grade inflammation and multisystem functional decline (2). This has led to growing interest in the gut as a potential therapeutic target for promoting healthier aging trajectories. The gut-brain axis and the gut-muscle axis, therefore, provide a useful framework for understanding how intestinal microbes may influence function in later life (3, 4). The gut-brain axis describes a bidirectional communication network involving microbial metabolites, immune mediators, endocrine pathways, and neural signaling, through which the gut microbiota may influence neuroinflammation, brain function, and cognition (3, 5). In parallel, the gut-muscle axis posits that intestinal dysbiosis may contribute to impaired muscle mass, strength, and performance through mechanisms including inflammation, altered nutrient handling, and disrupted metabolic homeostasis (4). Together, these interconnected axes suggest that the gut microbiota may influence two major functional domains in older age: cognition and muscle function. These domains are highly relevant to healthy aging, as cognitive decline contributes to dependency, reduced self-care capacity, and vulnerability to neurodegenerative disease, whereas sarcopenia compromises muscle strength, mobility, physical performance, and the ability to carry out activities of daily living (6, 7).

Against this background, microbiota-directed interventions have gained increasing attention. However, the present meta-analysis is not intended to cover all possible microbiota-modulating strategies. Instead, it focuses specifically on live microorganism-based interventions, namely probiotics, synbiotics, and fecal microbiota transplantation (FMT), when eligible studies meeting the review criteria were available. According to current international consensus definitions, probiotics are live microorganisms that, when administered in adequate amounts, confer a health benefit on the host, whereas synbiotics are mixtures comprising live microorganisms and selectively utilized substrates that confer a health benefit on the host (8, 9). Although FMT is more complex, it follows the same broad therapeutic logic by transferring a living microbial ecosystem to reshape gut ecology more extensively (10). This narrower focus is important because the broader microbiome field also includes dietary fibers, polyphenols, exercise, drugs, postbiotics, and other approaches that may influence gut ecology indirectly or through non-living products (11). Restricting the scope to live microorganism-based interventions, therefore, avoids grouping substantially different strategies under a single umbrella and allows a more clinically interpretable synthesis.

This focus is also timely because the available literature remains limited and heterogeneous. Recent systematic reviews and meta-analyses suggest that probiotic supplementation may improve cognitive performance, particularly in adults with mild cognitive impairment (MCI) or Alzheimer’s disease (AD), but substantial variability across study designs, formulations, treatment duration, and outcome measures limits firm conclusions (12, 13). Moreover, although emerging reviews in older adults suggest that probiotics and synbiotics may improve muscle strength and physical performance, evidence on muscle-related outcomes remains limited (14).

Therefore, the novelty of the present meta-analysis lies in jointly evaluating cognitive and muscle-related outcomes within a single framework of live microorganism-based interventions in aging adult populations, operationalized as study samples with a mean participant age ≥60 years. Whereas previous literature has generally assessed these domains separately (15, 16), the present study examines whether probiotics, synbiotics, and FMT may influence two major components of functional ability in aging. Hence, the aim of this meta-analysis was to evaluate human studies using live microorganism-based interventions, including probiotics, synbiotics, and eligible FMT studies, in aging adult populations, focusing on their effects on cognitive and muscle-related outcomes.

## 2 Methods

### 2.1 Study design

This systematic review and meta-analysis was conducted in accordance with the Preferred Reporting Items for Systematic Reviews and Meta-Analyses (PRISMA) 2020 statement (17) (Figure 1). Eligibility criteria requiring a mean participant age of ≥60 years were applied during title/abstract screening and full-text assessment, rather than as a universal database filter. This criterion was applied at the study-population level and therefore did not require every individual participant in each trial to be aged ≥60 years. The present systematic review includes both a qualitative synthesis of all eligible studies (Table 1) and a quantitative synthesis (meta-analysis) when at least a subset of studies reported sufficiently comparable outcome data (Table 2). The protocol was prospectively registered in the International Prospective Register of Systematic Reviews (PROSPERO; registration no. CRD420251065993; https://www.crd.york.ac.uk/PROSPERO/view/CRD420251065993).

**Figure 1:**
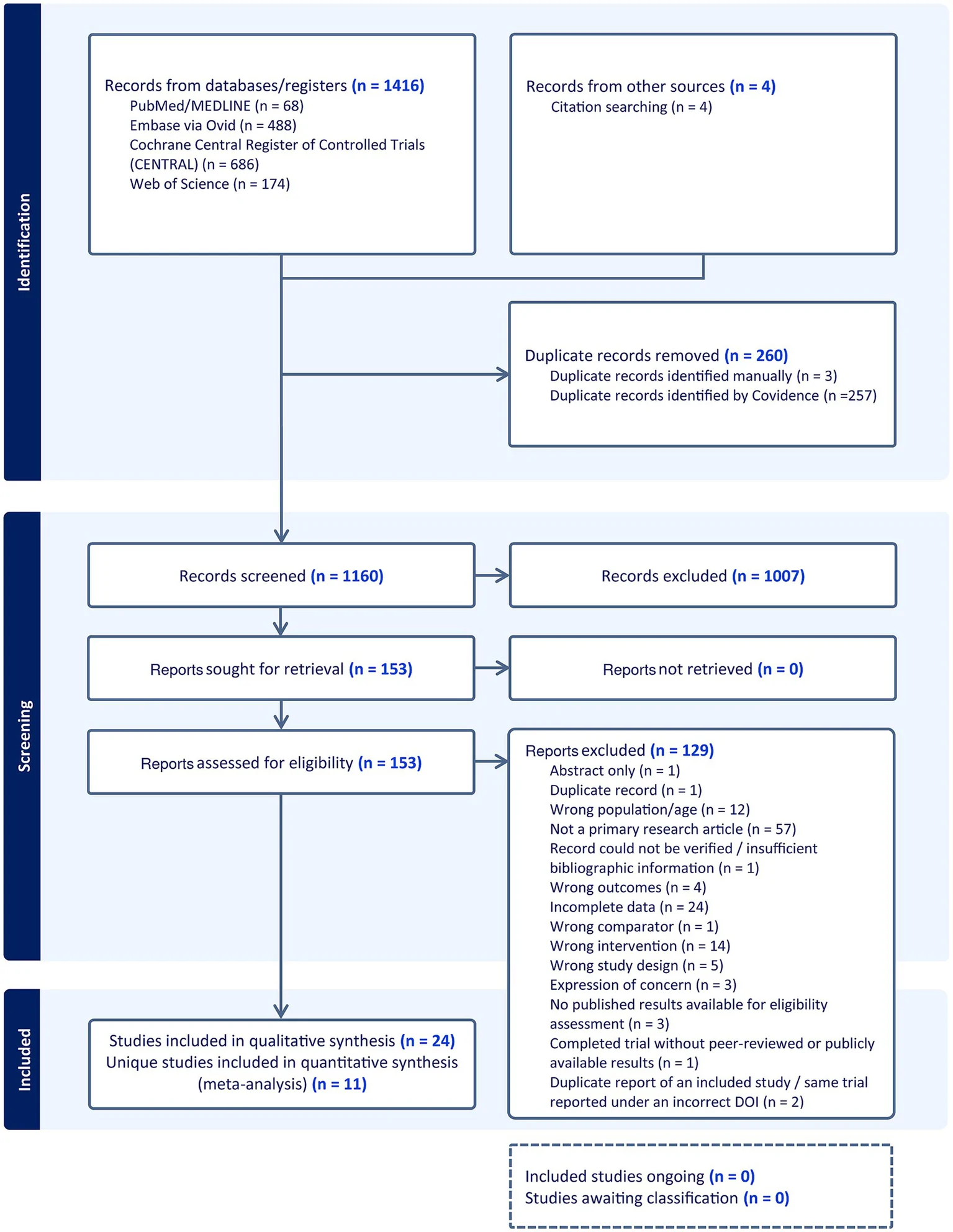
PRISMA flow diagram of study selection. The diagram shows the identification, screening, eligibility, and inclusion process. A total of 1,420 records were identified: 1,416 from databases and registers and four through citation searching. After removal of 260 duplicate records, 1,160 records were screened. A total of 153 full-text reports were assessed for eligibility, of which 129 were excluded and 24 studies were included in the qualitative synthesis. Eleven unique studies contributed to at least one quantitative synthesis, representing 12 study–outcome contributions and 14 intervention comparisons.

**Table 1:** Characteristics of the studies included in the qualitative synthesis.

Author, year, country	Intervention (type and duration)	Target population	Administration route	Design/follow-up	Study population (n)	Age (mean and SD)	Cognitive/muscle-related assessments
Studies evaluating cognitive function							
Akbari et al. (2016), Iran (20)	Intervention: Probiotic *Lactobacillus acidophilus, Lactobacillus casei, Bifidobacterium bifidum*, and *Lactobacillus fermentum* (2 × 109 CFU/g for each) Placebo: Not reported	Older adults aged 60–95 years with Alzheimer’s disease	Oral (200 mL/day probiotic milk)	RCT, double-blind Follow-up: 12 weeks.	Intervention: 30 Placebo: 30 TOTAL: 60	Intervention: 77.7 (2.6) Placebo: 82.0 (1.7) TOTAL: 78.1 (5.1)	MMSE
Akhgarjand et al. (2022), Iran (21)	Intervention 1: Probiotic *L. rhamnosus* HA-114 (1 × 10^15^ CFU, 2 × day) Intervention 2: *B. longum* R0175 (1 × 10^15^ CFU, 2 × day) Placebo: Xylitol, maltodextrin, malic acid (2×/day)	Adults aged 50–90 years with mild or moderate Alzheimer’s disease	Oral (2 capsules per day)	Multicenter RCT, parallel design, three arms, double-blind, placebo-controlled Follow-up: 12 weeks.	Intervention 1: 30 Intervention 2: 30 Placebo: 30 TOTAL: 90	Intervention 1: 67.9 (7.8) Intervention 2: 67.9 (7.9) Placebo: 67.8 (7.9) TOTAL: 67.9 (7.9)	MMSE CFT ADL IADL
Asaoka et al. (2022), Japan (22)	Intervention: Probiotic *Bifidobacterium breve* MCC1274 (2 × 10^10^ CFU/d) Placebo: maize-starch	Older adults aged 65–89 years with suspected MCI	Oral (1 sachet per day)	RCT, double-blind, placebo-controlled, parallel-group Follow-up: 24 weeks	Intervention: 55 Placebo: 60 TOTAL: 115	Intervention: 77.2 (5.8) Placebo: 78.9 (4.3) TOTAL: 78.1 (5.1)	ADAS-Jcog MMSE Modified MMSE
Azuma et al. (2023), Japan (23)	Intervention: Synbiotic *B. animalis subsp. lactis* GCL2505 (1 × 10^10^ CFU) + inulin (2 g/d) Placebo: the same dairy drink without synbiotics	Community-dwelling Japanese men and women, (50–80 years), with MCI but no dementia.	Oral (dairy drink)	RCT, double-blind, placebo-controlled Follow-up: 12 weeks	Intervention: 32 Placebo: 35 TOTAL: 67	Intervention: 64.6 (7.1) Placebo: 62.7 (6.9) TOTAL: 63.7 (6.0)	Cognitrax *Only on baseline:* MMSE-J and MoCA-J
Baek et al. (2025), Korea (24)	Intervention: Probiotic *Lactobacillus delbrueckii subsp. lactis* CKDB001 (5 × 10^9^ CFU/day) Placebo: microcrystalline cellulose and magnesium stearate without probiotic	Adults aged 55–80 years with MCI	Oral (two capsules of 300 mg)	RCT, double-blind, placebo-controlled, multicenter trial Follow-up: 12 weeks	Intervention: 48 Placebo: 48 TOTAL: 96	Intervention: 67.3 (4.6) Placebo: 67.4 (4.9) TOTAL: 67.4 (4.8)	ADAS-cog-13 DST TMT Stroop test
Bartos et al. (2023), Czechia (25)	Intervention: Synbiotic: human-derived *Streptococcus thermophilus GH, Streptococcus salivarius GH NEXARS, Lactobacillus plantarum GH,* and *Pediococcus pentosaceus* GH (10^6^ CFU/day) plus 2 fiber tablets/day Placebo: tablet composed of semi-coarse wheat flour, starch, maltodextrin, and magnesium stearate; the fiber placebo tablet was composed of cellulose, maltodextrin, and stearate magnesium.	Community-dwelling adults aged 55–80 years with normal cognition or MCI	Oral (1 probiotic/placebo tablet along with 2 fiber/placebo fiber tablets once a day)	RCT, double-blind, placebo-controlled trial with a cross-over Follow-up: 3 months each group	Intervention: 47 Placebo: 44 TOTAL: 91	Intervention: 69.0 (7.0) Placebo: 71.5 (6.0) TOTAL: 70.0 (7.0)	CDT ALBA ABACO PICNIR RAVLT TMT WAIS-III FAQ-CZ
Chung et al. (2014), Korea (26)	Intervention 1: Fermented milk with a probiotic, *L. helveticus* IDCC3801 (LHFM) (125 mg of LHFM) Intervention 2: Fermented milk with a probiotic, *L. helveticus* IDCC3801 (LHFM) (250 mg of LHFM) Intervention 3: Fermented milk with a probiotic, *L. helveticus* IDCC3801 (LHFM) (500 mg of LHFM) Placebo: the same preparation method without LHFM	Healthy older adults aged 60–75 years	Oral (four tablets per day, which were taken at same time once a day, with 500, 1,000, and 2000 mg as daily dose)	RCT, double-blind, placebo-controlled Follow-up: 12 weeks	Intervention 1: 10 Intervention 2: 7 Intervention 3: 9 Placebo: 10 TOTAL: 36	Intervention 1: 64.5 (2.2) Intervention 2: 64.4 (4.5) Intervention 3: 66.6 (5.0) Placebo: 64.5 (4.8) TOTAL: 65.0 (4.1)	DST VLT RVIP *Only on baseline:* MMSE-K
Eastwood et al. (2025), United Kingdom (27)	Intervention: Multistrain probiotic containing nine bacterial strains: *Bifidobacterium lactis* W51, *B. lactis* W52, *Lactobacillus acidophilus* W37, *Ligilactobacillus salivarius* W24, *Lacticaseibacillus casei* W56, *Bifidobacterium bifidum* W23, *Levilactobacillus brevis* W63, *Lactococcus lactis* W19, and *Lactococcus lactis* W58 (5 × 10^9^ CFU/day), containing maize starch, maltodextrin, vegetable protein, and potassium chloride Placebo: identical to the intervention without the bacterial strains	Healthy older adults aged 65–80 years	Oral (1 sachet per day)	RCT, double-blind, cross-over, placebo-controlled Follow-up: 8 weeks	Intervention: 15 Placebo: 15 TOTAL: 30	Intervention: 71.7 (4.5) Placebo: 70.7 (3.9) TOTAL: 71.2 (4.2)	RAVLT CBTT Go/no-go Task *Only on baseline:* MoCA
Fei et al. (2023), China (28)	Intervention: Multistrain probiotic formulation (2 g/day; >2 × 10^10^ CFU/g) containing 18 bacterial strains: *Lactobacillus plantarum* BioF-228 and CN2018, *Lactococcus lactis* BioF224 and LY-66, *Bifidobacterium lactis* CP-9 and HN019, *Lactobacillus rhamnosus* Bv-77 and HN001, *Lactobacillus johnsonii* MH-68, *Lactobacillus paracasei* MP137 and GL-156, *Lactobacillus salivarius* AP-32, *Lactobacillus acidophilus* TYCA06, *Bifidobacterium animalis* BB-115, *Lactobacillus casei* CS-773, *Lactobacillus reuteri* TSR332, *Lactobacillus fermentum* TSF331, and *Bifidobacterium infantis* BLI-02. Placebo: 2 g starch capsule.	Older adults (>60 years old) with MCI	Oral (2 g capsule daily)	RCT, placebo-controlled Follow-up: 12 weeks	Intervention: 20 Placebo: 20 TOTAL: 40	Intervention: 68.0 (5.1) Placebo: 69.2 (7.0) TOTAL: 68.6 (6.1)	MMSE MoCA
Hwang et al. (2019), Korea (29)	Intervention: Probiotic with 800 mg/day of *Lactobacillus* genus. Placebo: made of cellulose.	Adults aged 55–85 years with MCI	Oral (2 capsules once a day)	RCT, double-blind, placebo-controlled Follow-up: 12 weeks	Intervention: 50 Placebo: 50 TOTAL: 100	Intervention: 75.3 (9.8) Placebo: 76.4 (9.6) TOTAL: 75.9 (9.7)	VLT ACPT DST
Kim et al. (2021), Korea (30)	Intervention: *Bifidobacterium bifidum* BGN4 and *Bifidobacterium longum* BORI (1 × 10^9^ CFU) in soybean oil Placebo: capsule contained 500 mg of soybean oil only.	Older adults (>65 years) with MCI	Oral (2 capsules twice a day)	Multicenter RCT, double-blind, placebo-controlled Follow-up: 12 weeks	Intervention: 27 Placebo: 26 TOTAL: 53	Intervention: 71.0 (0.1) Placebo: 72.0 (0.0) TOTAL: 71.0 (0.5)	CERAD-K *Only on baseline:* MMSE.
Kobayashi et al. (2019), Japan (31)	Intervention: *Bifidobacterium breve* A1 (>2.0 × 10^10^ CFU) Placebo: corn starch with no *Bifidobacteria*	Adults aged 50–80 years with subjective memory complaints	Oral (2 capsules daily)	RCT, double-blind, placebo-controlled Follow-up: 12 weeks	Intervention: 59 Placebo: 58 TOTAL: 117	Intervention: 61.5 (6.8) Placebo: 61.6 (6.4) TOTAL: 61.6 (6.6)	MMSE RBANS
Lazou-Ahren et al. (2025), Sweden (32)	Intervention 1: Probiotic *Lactobacillus plantarum* HEAL9 Intervention 2: Probiotic *Lactobacillus plantarum* HEAL9 in combination with lyophilized mixture of freeze-dried blackberries and black currants (6 g freeze-dried powder, consisting of 3 g of each berry, derived from 43 g of fresh berries). Placebo: maltodextrin powder	Healthy older adults (>70 years) with systemic low-grade inflammation	Oral (powder with 10 g per sachet as the daily dose consumed after mixing with sour milk or pouring over the breakfast flakes)	RCT, double-blind, placebo-controlled Follow-up: 12 weeks	Intervention 1: 21 Intervention 2: 21 Placebo: 22 TOTAL: 64	Intervention 1: 73.2 (1.8) Intervention 2: 73.0 (1.9) Placebo: 73.1 (2.1) TOTAL: 73.1 (2.0)	TMT
Louzada and Ribeiro (2020), Brazil (33)	Intervention: Synbiotic containing fructooligosaccharide (6 g), *Lactobacillus paracasei* (10^9^–10^8^ CFU), *Lactobacillus rhamnosus* (10^9^–10^8^ CFU), *Lactobacillus acidophilus* (10^9^–10^8^ CFU), and *Bifidobacterium lactis* (10^9^–10^8^ CFU) Placebo: maltodextrin	Healthy older adults aged 65–90 years classified as pre-frail	12 g daily (6 g morning + 6 g night)	RCT, double-blind, placebo-controlled Follow-up: 24 weeks	Intervention: 25 Placebo: 24 TOTAL: 49	Intervention: 77.2 (1.3) Placebo: 77.0 (1.3) TOTAL: 77.1 (1.3)	MMSE
Mehmandoust et al. (2024), Iran (34)	Intervention: Synbiotic formulation containing seven probiotic strains (10^9^ CFU) including *Lactobacillus rhamnosus*, *L. bulgaricus*, *L. casei*, *L. acidophilus*, *Bifidobacterium breve*, *B. longum*, and *Streptococcus thermophilus*, combined with fructooligosaccharides (FOS) as a prebiotic. Placebo: maltodextrin	Older adults (> 60 years) with Alzheimer’s disease	Oral (two capsules daily)	RCT, double-blind, placebo-controlled Follow-up: 12 weeks	Intervention: 25 Placebo: 27 TOTAL: 52	Intervention: 77.0 (8.5) Placebo: 77.3 (8.4) TOTAL: 77.2 (8.5)	MMSE
Ruiz-Gonzalez et al. (2025), Spain (35)	Intervention: Probiotic (3.3 billion CFU *Lactobacillus rhamnosus* and *Bifidobacterium*) Placebo: potato starch	Healthy adults (over 55 years)	Oral (one capsule daily)	RCT, double-blind, placebo-controlled crossover trial Follow-up: 10 weeks	Intervention: 16 Placebo: 17 TOTAL: 33	Intervention: Not reported Placebo: Not reported TOTAL: 65.9 (6.2)	MMSE PEBL Digit task Tower of London Corsi task Wisconsin Card Sorting Test Stroop task Trail Making test Go/No Go task Iowa Gambling
Sanborn et al. (2020), USA (36)	Intervention: Probiotic *Lactobacillus rhamnosus* GG (2 capsules × 10 billion CFU/day) Placebo: microcrystalline cellulose	Middle aged and older adults aged 52–75 years	Oral (two capsules daily)	RCT, double-blind, parallel, placebo-controlled trial Follow-up: 3 months	Intervention: 76 Placebo: 68 TOTAL: 144	Intervention: 64.6 (5.6) Placebo: 64.1 (5.3) TOTAL: 64.3 (5.5)	NIH Toolbox Cognition Battery
Shi et al. (2023), China (37)	Intervention: Probiotic *Bifidobacterium longum* BB68S (5 × 10^10^ CFU/sachet) Placebo: maltodextrin powder without probiotics	Healthy older adults aged 60–75 years	Oral (one sachet a sachet after lunch or dinner daily)	RCT, double-blind, placebo-controlled Follow-up: 8 weeks	Intervention: 25 Placebo: 25 TOTAL: 50	Intervention: 64.1 (3.4) Placebo: 64.5 (3.8) TOTAL: 64.3 (3.6)	MoCA RBANS
Wang et al. (2021), China (38)	Intervention: Probiotic *Bifidobacterium longum* (>1.0 × 10^7^ CFU/capsule), *Lactobacillus acidophilus* (>1.0 × 10^7^ CFU/capsule), and *Enterococcus faecalis* (>1.0 × 10^7^ CFU/capsule). Placebo: lactose	Older adults (60–90 years) undergoing elective hip or knee replacement surgery or colorectal cancer resection surgery	Oral (0.84 g twice daily)	RCT, double-blind, placebo-controlled Follow-up: 8 weeks	Intervention: 59 Placebo: 61 TOTAL: 120	Intervention: 69.6 (5.7) Placebo: 70.6 (6.9) TOTAL: 70.3 (6.2)	MMSE
Xiao et al. (2020), Japan (39)	Intervention: Probiotic *Bifidobacterium breve* A1 (>1.0 × 10^10^ CFU) Placebo: maize starch	Adults aged 50–79 years with suspected MCI	Oral (two capsules daily)	RCT, double-blind, placebo-controlled Follow-up: 16 weeks	Intervention: 59 Placebo: 61 TOTAL: 120	Intervention: 61.3 (7.7) Placebo: 60.9 (6.9) TOTAL: 61.1 (7.3)	RBANS JMCIS *Only on baseline:* MMSE
Studies evaluating muscle-related function							
Lee et al. (2021), Taiwan (40)	Intervention 1: *Lactobacillus plantarum* TWK10 low dose 2 × 10^10^ CFU/day Intervention 2: *Lactobacillus plantarum* TWK10 high dose 6 × 10^10^ CFU/day Placebo: capsules containing only maltodextrin and microcrystalline cellulose	Adults aged 55–85 years with mild frailty (CFS 1–4)	Oral capsules (two per day)	RCT, placebo-controlled trial; three arms (placebo, TWK10-L, TWK10-H). Follow-up: 18 weeks	Intervention 1: 12 Intervention 2: 13 Placebo: 17 TOTAL: 42	Intervention 1: 77.8 (7.2) Intervention 2: 80.5 (9.4) Placebo: 75.2 (7.2) TOTAL: 77.6 (8.1)	HGS TUG ASM (DXA) Chair stand Gait speed
Lee et al. (2025), Taiwan (41)	Intervention 1: Live *Lactobacillus paracasei* PS23 (L-PS23; 2 × 10^10^ CFU/day). Intervention 2: Heat-treated *Lactobacillus paracasei* PS23 (HT-PS23; 2 × 10^10^ cells/day). Placebo: microcrystalline cellulose	Community-dwelling older adults aged 65–85 years	Oral (2 capsules per day)	RCT, double-blind, placebo-controlled; three groups Follow-up: 12 weeks	Intervention 1: 33 Intervention 2: 34 Placebo: 33 TOTAL: 100	Intervention 1: 69.8 (3.0) Intervention 2: 69.9 (3.3) Placebo: 69.3 (3.0) TOTAL: 69.7 (3.1)	HGS BIA TUG ISPT Chair stand Arm curl
Neto et al. (2013), Brazil (42)	Intervention: FOS + *L. paracasei, L. rhamnosus, L. acidophilus & B. lactis* (10^8^–10^9^ CFU) Placebo: maltodextrin	Older adults aged 60–75 years with ≥1 frailty criterion	Oral (powder sachets)	RCT, double-blind, placebo-controlled pilot Follow-up: 12 weeks	Intervention: 9 Placebo: 8 TOTAL: 17	Intervention: Not reported Placebo: Not reported TOTAL: 67.9 (4.5)	HGS BIA
Qaisar et al. (2024), Pakistan (43)	Intervention Multistrain probiotic formulation (Vivomixx® 112; 112 × 10^9^ CFU/day) containing *Bifidobacterium longum* DSM 24736, *Bifidobacterium breve* DSM 24732 and DSM 24737, *Streptococcus thermophilus* DSM 24731, *Lactobacillus* spp. DSM 24735, DSM 24730 and DSM 24733, and *Lactobacillus delbrueckii subsp. bulgaricus* DSM 24734. Placebo: inert capsule	Men ≥65 years with sarcopenia	Oral (1 capsule per day)	RCT, double-blind, placebo-controlled; Follow-up: 16 weeks	Intervention: 60 Placebo: 63 TOTAL: 123	Intervention: 73.0 (4.1) Placebo: 71.4 (3.9) TOTAL: 72.2 (4.0)	HGS Gait speed BIA

This table summarizes the main characteristics of the 24 placebo-controlled clinical trials included in the systematic review. For each study, the table reports the author and year, intervention and placebo, study population, route and regimen of administration, study design, follow-up duration, sample size, mean age, and cognitive or muscle-related outcomes assessed. Studies are grouped according to whether they evaluated cognitive or muscle-related function. RCT, randomized clinical trial; MCI, mild cognitive impairment; MMSE, Mini-Mental State Examination; ADL, Activities of Daily Living; IADL, Lawton Instrumental Activities of Daily Living; CFT, categorical verbal fluency test; ADAS-Jcog, Japanese version of Alzheimer’s Disease Assessment Scale – Cognitive Subscale (ADAS-cog); MoCA-J, Japanese version of the Montreal Cognitive Assessment (MoCA); CDT, Clock Drawing Test; ALBA, Amnesia Light and Brief Assessment; ABACO, Assessment Battery of Cognition; PICNIR, PICture Naming and Immediate Recall; RAVLT, Rey Auditory Verbal Learning Test; TMT, Trail Making Test; WAIS-III, Wechsler Adult Intelligence Scale III; FAQ-CZ, Functional Activities Questionnaire; CBTT, Corsi Block Tapping Task; TST, Task Switching Task; VLT, verbal learning test; ACPT, auditory continuous performance test; DST, digit span test; CERAD-K, Consortium to Establish a Registry for Alzheimer’s Disease–Korea; PEBL, Psychology Experiment Building Language Test Battery; JMCIS, Japanese version of the MCI Screen; CFS, Clinical Frailty Scale; RBANS, Repeatable Battery for the Assessment of Neuropsychological Status; HGS, handgrip strength; TUG, Timed Up and Go Test; ASM (DXA), appendicular skeletal muscle mass measured by dual-energy X-ray absorptiometry; BIA, bioelectrical impedance analysis; ISPT, Incremental Step-in-Place Test.

**Table 2:** Characteristics of studies contributing to the outcome-specific meta-analyses.

Author, year, health status	Intention-to-treat/Sample size	Inclusion	Exclusion	Baseline	Final
Studies evaluating cognitive function - MMSE					
Akbari et al. (2016) (20) Alzheimer’s disease	Yes/calculated	People with Alzheimer’s disease (60–95 years old) residing at the Golabchi (Kashan, Iran) and Sadeghyeh (Esfahan, Iran) Welfare Organizations between December 2015 and February 2016.	Patients with metabolic disorders, chronic infections and/or other clinically, consuming probiotic supplements within 6 weeks prior to the study, taking other probiotic yogurt, kefir, and other fermented foods.	*Total score* Intervention: 8.67 (1.44) Placebo: 8.47 (1.10)	*Total score* Intervention: 10.57 (1.64) Placebo: 8.00 (1.08)
Akhgarjand et al. (2022) (21) Alzheimer’s disease	No/calculated	Aged 50–90 years, ability to tolerate oral medication, and mild or moderate Alzheimer’s disease based on the Functional Assessment Staging Tool (FAST) score 4-6B.	Allergy to probiotic supplements, reluctance to continue cooperation, drastic changes in diet, inflammatory diseases that lead to long-term use (more than 2 weeks) of anti-inflammatory drugs, taking antibiotics, pre-probiotic or probiotic products, or multivitamin and mineral supplements during the intervention, having any kind of infection or another disease like COVID-19, changing the type of drug during the intervention, and enrollment in any other intervention trial.	*Total score* Intervention 1: 15.73 (3.89) Intervention 2: 15.50 (3.22) Placebo: 15.47 (3.03) *Orientation* Intervention 1: 6.37 (2.02) Intervention 2: 6.30 (1.60) Placebo: 5.87 (1.61) *Registration* Intervention 1: 2.87 (0.34) Intervention 2: 2.87 (0.34) Placebo: 2.83 (0.37) *Attention* Intervention 1: 0.17 (0.37) Intervention 2: 0.07 (0.25) Placebo: 0.17 (0.59) *Recall* Intervention 1: 1.03 (0.76) Intervention 2: 1.07 (0.74) Placebo: 1.50 (0.86) *Language* Intervention 1: 5.20 (1.15) Intervention 2: 5.07 (1.14) Placebo: 5.03 (1.06) *Visuospatial* Intervention 1: 0.10 (0.30) Intervention 2: 0.13 (0.34) Placebo: 0.07 (0.25)	*Total score* Intervention 1: 18.10 (4.45) Intervention 2: 18.67 (3.05) Placebo: 13.77 (3.38) *Orientation* Intervention 1: 6.97 (1.95) Intervention 2: 7.03 (1.45) Placebo: 5.17 (1.57) *Registration* Intervention 1: 2.90 (0.40) Intervention 2: 3.00 (0.001) Placebo: 2.57 (0.56) *Attention* Intervention 1: 0.6 (0.77) Intervention 2: 0.43 (0.62) Placebo: 0.13 (0.57) *Recall* Intervention 1: 2.07 (0.90) Intervention 2: 2.37 (0.76) Placebo: 1.03 (0.76) *Language* Intervention 1: 5.33 ± 1.09 Intervention 2: 5.47 ± 0.93 Placebo: 4.83 ± 1.02 *Visuospatial* Intervention 1: 0.20 (0.40) Intervention 2: 0.37 (0.49) Placebo: 0.03 (0.18)
Mehmandoust et al. (2024) (34) Alzheimer’s disease	No/calculated	Only patients with Alzheimer’s disease were selected for the study	Infection at the time of assessment; severe comorbidities; gastrointestinal diseases or surgery; metabolic, immunosuppressive, or psychiatric disorders; alcohol or drug abuse; use of antibiotics, probiotic supplements, or other drugs; or consumption of probiotic products in the previous 3 months. Patients who developed new conditions affecting cognition or physical function (e.g., delirium or stroke) during the study, or who required antibiotics, were also excluded.	*Total score:* Intervention: 16.6 (1.08) Placebo: 16.1 (1.04)	*Total score:* Intervention: 18.4 (1.1) Placebo: 16.8 (1.1)
Asaoka et al. (2022) (22) MCI	No/calculated	Age 65–89 years, satisfied with the clinical criteria of MCI (DSM-5), and the following three criteria: (i) Memory complaint by subject or family, (ii) MMSE scores between 22 and 26 (inclusive), and (iii) Clinical Dementia Rating (CDR) = 0.5; and written informed consent provided for study participation.	Parkinson’s or Huntington’s disease, normal pressure hydrocephalus, progressive supranuclear palsy, epilepsy, multiple sclerosis, cerebral infection, or subsequent complication caused by head trauma; history of major depression or bipolar disorder, alcohol or other substance abuse; findings of multiple infarction, brain tumor, or subdural hematoma; cognitive impairment due to deficiency of vitamin B12 or folate; neurosyphilis; thyroid function abnormalty; severe disease; heavy users of pharmaceutical products affecting the bowel movement regularity and health food, supplement; remarkable abnormality for blood pressure and blood tests; allergy for drugs and food; heavy smoker, drinker; users for Anti-dementia drugs, psychoactive drugs, severe diabetes mellitus treated with insulin; contraindications for MRI such as magnetic body or metal; participation in any other new drug study for AD or another new drug study.	*Total score* Intervention: 24.51 (1.23) Placebo: 24.62 (1.17) *Orientation* Intervention: 4.64 (0.56) Placebo: 4.6 (0.74) *Registration* Intervention: 3.00 (0.0) Placebo: 2.97 (0.18) *Recall* Intervention: 2.2 (0.87) Placebo: 2.17 (0.85) *Visuospatial* Intervention: 4.62 (0.56) Placebo: 4.62 (0.67) *Attention/Naming* Intervention: 2.0 (0.0) Placebo: 2.0 (2.0) *Construction* Intervention: 0.91 (0.29) Placebo: 0.88 (0.32)	*Total score* Intervention: 27.08 (2.42) Placebo: 27.25 (2.38) *Orientation* Intervention: 4.82 (0.52) Placebo: 4.6 (0.8) *Registration* Intervention: 2.98 (0.14) Placebo: 3.00 (0.0) *Recall* Intervention: 2.71 (0.64) Placebo: 2.65 (0.72) *Visuospatial* Intervention: 4.92 (0.27) Placebo: 4.81 (0.48) *Attention/Naming* Intervention: 2.0 (0.0) Placebo: 2.0 (2.0) *Construction* Intervention: 0.98 (0.14) Placebo: 0.97 (0.19)
Fei et al. (2023) (28) MCI	No/for convenience	Over 60 years of age; irrespective of sex; met the Peterson diagnostic criteria of MCI21; had no serious heart, lung, liver or kidney dysfunction; had no chronic disease exacerbation or seizure status; had no obvious visual or hearing impairment; understood the purpose and contents of this study; and agreed to sign the informed consent form.	Participation in other clinical studies and allergies to dairy products.	*Total score* Intervention: 21.75 (2.57) Placebo: 20.85 (2.25) *Orientation* Intervention: 9.30 (0.66) Placebo: 9.50 (0.76) *Registration (Memory)* Intervention: 2.90 (0.45) Placebo: 2.90 (0.31) *Recall* Intervention: 0.90 (0.79) Placebo: 0.65 (0.74) *Attention and calculation* Intervention: 2.20 (1.32) Placebo: 1.75 (0.72) *Language* Intervention: 6.40 (1.04) Placebo: 6.05 (1.31)	*Total score* Intervention: 24.75 (2.47) Placebo: 20.95 (1.36) *Orientation* Intervention: 9.80 (0.41) Placebo: 9.70 (0.47) *Registration (Memory)* Intervention: 2.90 (0.31) Placebo: 2.90 (0.31) *Recall* Intervention: 1.95 (0.76) Placebo: 0.70 (0.47) *Attention and calculation* Intervention: 3.55 (1.54) Placebo: 1.60 (0.60) *Language* Intervention: 6.55 (1.19) Placebo: 6.05 (0.89)
Kobayashi et al. (2019) (31) Subjective memory complaints	No/for convenience	Age, 50–80 years; and MMSE score, 22–27.	Diagnosed with dementia; current or history of psychiatric disorders, drug dependence, drug abuse or alcoholism; on diet and exercise therapy under doctor’s recommendation; history of liver disease or serious renal, endocrine, cardiovascular, gastrointestinal, pulmonary, haematological and metabolic diseases or those undertaking medication or treatment for such diseases; history of major surgery involving gastrectomy, gastrointestinal suture surgery, bowel resection and digestive system; serious allergy to medicine or food; participated in other clinical tests within the past month; irregular working hours, such as night shifts; and judged inadequate by the study physician.	*Total score* Intervention: 26.10 (0.96) Placebo: 26.31 (0.90) *Orientation* Intervention: 4.59 (0.59) Placebo: 4.69 (0.50) *Registration* Intervention: 2.98 (0.13) Placebo: 2.97 (0.18) *Recall* Intervention: 1.66 (0.98) Placebo: 1.67 (0.85) *Language* Intervention: 7.34 (0.69) Placebo: 7.43 (0.68) *Visuospatial* Intervention: 4.17 (0.79) Placebo: 4.24 (0.66) *Attention/Calculation* Intervention: 4.44 (0.75) Placebo: 4.34 (0.83)	*Total score* Intervention: 27.73 (1.62) Placebo: 28.07 (1.62) *Orientation* Intervention: 4.88 (0.32) Placebo: 4.93 (0.26) *Registration* Intervention: 3.00 (0.0) Placebo: 3.00 (0.0) *Recall* Intervention: 1.76 (1.09) Placebo: 2.10 (1.00) *Language* Intervention: 7.76 (0.43) Placebo: 7.83 (0.42) *Visuospatial* Intervention: 4.61 (0.53) Placebo: 4.60 (0.59) *Attention/Calculation* Intervention: 4.73 (0.48) Placebo: 4.60 (0.77)
Studies evaluating cognitive function - RBANS					
Kobayashi et al. (2019) (31) Subjective memory complaints	No/for convenience	Age, 50–80 years; and MMSE score, 22–27.	Diagnosed with dementia; current or history of psychiatric disorders, drug dependence, drug abuse or alcoholism; on diet and exercise therapy under doctor’s recommendation; history of liver disease or serious renal, endocrine, cardiovascular, gastrointestinal, pulmonary, haematological and metabolic diseases or those undertaking medication or treatment for such diseases; history of major surgery involving gastrectomy, gastrointestinal suture surgery, bowel resection and digestive system; serious allergy to medicine or food; participated in other clinical tests within the past month; irregular working hours, such as night shifts; and judged inadequate by the study physician.	*Total score* Intervention: 41.64 (17.42) Placebo: 49.07 (14.24) *Immediate memory* Intervention: 44.44 (13.10) Placebo: 49.05 (9.19) *Visuospatial*/*constructional* Intervention: 50.21 (12.88) Placebo: 52.23 (9.58) *Language* Intervention: 46.42 (11.58) Placebo: 48.72 (11.68) *Attention* Intervention: 47.54 (14.76) Placebo: 51.23 (11.66) *Delayed memory* Intervention: 39.80 (16.52) Placebo: 46.90 (13.05)	*Total score* Intervention: 46.91 (15.30) Placebo: 53.72 (13.83) *Immediate memory* Intervention: 44.82 (13.11) Placebo: 46.98 (10.95) *Visuospatial*/*constructional* Intervention: 49.57 (12.15) Placebo: 51.97 (9.52) *Language* Intervention: 52.07 (11.30) Placebo: 56.79 (11.24) *Attention* Intervention: 51.98 (13.15) Placebo: 56.22 (10.87) *Delayed memory* Intervention: 43.34 (12.32) Placebo: 48.32 (12.10)
Shi et al. (2023) (37) Healthy older adults	Yes/calculated	Aged 60–75 years at the time of screening; and with healthy cognitive function judged by the Montreal Cognitive Assessment (MoCA) screening tool.	Obvious cognitive decline, or diagnosis with Alzheimer’s disease, mild cognitive impairment, etc.; hearing, visual, or communication disabilities or difficulties, incapable of living independently, or surgical history of digestive resection; history or clinical trace of nervous system disease or psychosis; history of serious diseases, such as those of the heart, liver, kidney, or hematopoietic system; use of the test- related product (other probiotic or antibiotics, ease or psychosis; (iv) history of serious diseases, such as those of the heart, liver, kidney, anti-inflammatory medications, gastrointestinal medicine) within a short period; unable to eat the test product according to the regulations, and participation in other clinical studies.	*Total score* Intervention: 37.38 (3.66) Placebo: 37.18 (3.35) *Immediate memory* Intervention: 32.20 (5.69) Placebo: 31.56 (5.03) *Visuospatial*/*constructional* Intervention: 30.68 (5.13) Placebo: 30.56 (4.08) *Language* Intervention: 30.36 (4.39) Placebo: 29.76 (3.56) *Attention* Intervention: 55.20 (9.80) Placebo: 55.44 (9.99) *Delayed memory* Intervention: 38.44 (4.54) Placebo: 38.56 (4.91)	*Total score* Intervention: 44.34 (3.24) Placebo: 40.39 (3.43) *Immediate memory* Intervention: 40.12 (4.35) Placebo: 35.28 (5.09) *Visuospatial*/*constructional* Intervention: 35.48 (2.35) Placebo: 33.40 (3.33) *Language* Intervention: 33.64 (3.87) Placebo: 32.08 (3.66) *Attention* Intervention: 66.64 (8.68) Placebo: 59.56 (10.34) *Delayed memory* Intervention: 45.84 (4.17) Placebo: 41.64 (5.10)
Xiao et al. (2020) (39) MCI	Yes/calculated	Age 50–79 years with Japanese nationality, MMSE score 22 or more.	Diagnosed with dementia; under exercise therapy or dietetic therapy; allergy for test food; history of medicine or alcohol dependence syndrome; history of mental illness or sleep disturbance; on a night-shift or is a shift worker; lifestyle is hugely irregular; an unbalanced diet; being treated or history of serious diseases; use of health foods, supplements, and medicines that may affect cognitive function; history of significant surgery involving gastrectomy, gastrointestinal surgery, bowel resection, and digestive system (excluding appendicitis); participation in other clinical studies within the past 3 months or who is planning to participate in other clinical studies during the current study; blood draw of 200 mL within the past 1 month or 400 mL within the past 3 months; cannot keep the daily records; judged ineligible based on the screening data or by the study physician.	*Total score* Intervention: 30.5 (10.6) Placebo: 32.4 (7.5) *Immediate memory* Intervention: 36.9 (10.5) Placebo: 36.4 (8.4) *Visuospatial/Constructional* Intervention: 32.1 (13.2) Placebo: 34.4 (14.4) *Language* Intervention: 49.9 (10.7) Placebo: 47.3 (7.8) *Attention* Intervention: 45.7 (11.0) Placebo: 49.2 (10.0) *Delayed memory* Intervention: 31.1 (12.0) Placebo: 31.1 (12.3)	*Total score* Intervention: 47.9 (13.4) Placebo: 38.3 (13.0) *Immediate memory* Intervention: 48.2 (11.2) Placebo: 38.7 (9.9) *Visuospatial/Constructional* Intervention: 46.2 (10.0) Placebo: 35.8 (13.5) *Language* Intervention: 54.2 (8.1) Placebo: 50.1 (8.8) *Attention* Intervention: 51.1 (10.2) Placebo: 53.3 (11.8) *Delayed memory* Intervention: 45.6 (14.2) Placebo: 34.6 (13.5)
Studies evaluating muscle-related function					
Lee et al. (2021), Taiwan (40) Clinical Frailty	No/calculated	Aged 55–85 years. Clinical Frailty Scale grade 1–4	Subject with Stroke; hypertension; exercise contraindications confirmed by the attending physician; use of additional probiotics, prebiotics, fermented products (yogurt or cheese), or antibiotics during the study.	*Hand Grip Strength (kg)* Intervention 1: 19.6 (5.8) Intervention 2: 18.3 (5.7) Placebo: 17.9 (6.1)	*Hand Grip Strength (kg)* Intervention 1: 19.5 (3.5) Intervention 2: 20.6 (6.2) Placebo: 17.6 (5.1)
Neto et al. (2013), Brazil (42) Frailty	No/for convenience	Aged 60–75 years, free-living, fulfilled one frailty criterion (low handgrip strength).	Participants should not have reported, during the 3 months before the experiment, acute or chronic inflammatory gastrointestinal disease or the use of antibiotics, prebiotics, probiotics, synbiotics, laxatives, or antidiarrheal medication.	*Hand Grip Strength (N)* Intervention: 15.0 (5.2) Placebo: 15.9 (2.7)	*Hand Grip Strength (N)* Intervention: 15.7 (5.3) Placebo: 17.2 (3.9)
Qaisar et al. (2024), Pakistan (43) Sarcopenia	No/calculated	Men ≥ 65 years; diagnosed with sarcopenia according to EWGSOP2 criteria (HGS < 27 kg and SMI < 7 kg/m^2^); no uncontrolled chronic diseases.	Use of antibiotics and/or probiotics in the last 6 weeks; musculoskeletal disorders; prolonged bedrest; malignancies; recent surgeries; cardiac pacemakers; uncontrolled chronic diseases with reduced ambulation	*Hand Grip Strength (kg)* Intervention: 23.7 (2.9) Placebo: 23.9 (2.3)	*Hand Grip Strength (kg)* Intervention: 25.9 (3.9) Placebo: 23.5 (3.0)

This table contains 12 study–outcome contributions from 11 unique studies, generating 14 intervention comparisons. Six studies contributed to the MMSE analysis (seven comparisons), three to the RBANS analysis (three comparisons), and three to the handgrip-strength analysis (four comparisons). Kobayashi et al. is presented under both MMSE and RBANS because the study contributed to both outcome-specific analyses. Akhgarjand et al. and Lee et al. each contributed two active intervention comparisons. Ruiz-Gonzalez et al. and Wang et al. were retained in the qualitative synthesis (Table 1) but did not contribute to the quantitative synthesis. MCI, mild cognitive impairment; MMSE, Mini-Mental State Examination; RBANS, Repeatable Battery for the Assessment of Neuropsychological Status.

### 2.2 Search strategy

A comprehensive literature search was conducted in PubMed/MEDLINE, Embase via Ovid, the Cochrane Central Register of Controlled Trials (CENTRAL), and Web of Science from January 1980 to May 2025, with a manual update performed in December 2025. Because the concept of sarcopenia was first introduced in 1989, the search was initiated in 1980 to ensure that potentially relevant early studies were not missed. The search strategy combined controlled vocabulary terms, including MeSH and Emtree terms where applicable, and free-text terms across three main domains: live microorganism-based interventions, aging-related populations, and cognitive or muscle-related outcomes. These domains were combined using Boolean operators and adapted to the syntax of each database. Searches were restricted to human studies and English-language publications when database filters allowed. Eligibility criteria requiring a mean participant age of ≥60 years were applied during screening and full-text assessment rather than as a universal database filter. The complete database-specific search strategies, Boolean combinations, filters, search dates, and number of records retrieved are provided in Supplementary Table S1.

### 2.3 Eligibility criteria

Studies were included if they met the following criteria: (i) the study population consisted of aging adults, operationalized at the study level as a sample with a mean participant age of ≥60 years; (ii) the intervention consisted of a live microorganism-based intervention, including probiotics, synbiotics, or fecal microbiota transplantation (FMT); (iii) the study was a randomized controlled trial with a placebo or control comparator; (iv) the study reported cognitive and/or muscle-related outcomes; and (v) the article was written in English. Studies were excluded if they: (i) included relevant co-interventions that could confound attribution of effects to the microbial intervention; (ii) did not report relevant outcomes; (iii) enrolled predominantly younger populations, defined as studies with a mean participant age <60 years or, when age-distribution data were available, studies in which more than 50% of participants were younger than 60 years; or (iv) were not primary research articles. FMT studies were considered eligible in principle but were excluded when the target population, primary clinical condition, co-interventions, or outcomes were not aligned with aging-related cognitive or muscle-related function.

### 2.4 Study selection

All records retrieved from the databases were exported and merged using EndNote version 21 and subsequently imported into Covidence (Veritas Health Innovation, Melbourne, Australia) for duplicate removal and screening. Study selection was conducted in two stages: (1) title and abstract screening and (2) full-text review. Each record was independently assessed by two reviewers working in paired teams (FS-O and VR; NV and PA-H). Disagreements were resolved through discussion and, when necessary, consultation with a senior reviewer (ST-V and GJ). The study selection process is summarized in the PRISMA flow diagram (Figure 1).

### 2.5 Data extraction

Data extraction was performed independently by two reviewers using a standardized extraction form. Data were extracted from all studies included in the qualitative synthesis. The following information was collected: (i) study characteristics (authors, year, country); (ii) participant characteristics (age, sample size, clinical condition); (iii) intervention details (type, dose, duration, and route of administration); (iv) comparator characteristics; (v) outcomes assessed; and (vi) main findings. For studies reporting microbiome-related outcomes, we additionally extracted the analytical approach; post-intervention changes in microbial diversity, composition, or targeted bacterial abundance; evidence of recovery, persistence, or engraftment of the administered microorganisms; and reported associations between microbiological changes and cognitive or muscle-related outcomes. When microbiome findings from an included trial were reported in a complementary publication, these data were linked to the corresponding trial without counting it as an additional study. For the quantitative synthesis, numerical outcome data were additionally extracted when studies reported sufficiently comparable measures. Any discrepancies were resolved through consensus with a senior reviewer. Studies could remain eligible for the qualitative synthesis while being excluded from an outcome-specific meta-analysis when their design, population, outcome data, or effect estimates were not compatible with the planned pooled analysis.

### 2.6 Outcomes

Primary outcomes were validated measures of cognitive function and muscle-related function. For the quantitative synthesis, outcomes were pooled only when a sufficient number of studies reported comparable data (three studies or more). This was the case for MMSE, RBANS, and handgrip strength. Other cognitive and muscle-related outcomes, including gait speed, muscle mass, and additional neuropsychological questionnaires, were synthesized narratively. Functional questionnaires such as ADL, IADL, and FAQ-CZ, as well as physical-performance and body-composition outcomes, were considered in the qualitative synthesis but were not pooled because they were reported inconsistently or by too few studies using comparable post-intervention data.

### 2.7 Risk of bias assessment

Methodological quality was assessed using the PEDro scale across all 24 placebo-controlled randomized trials included in the qualitative synthesis. The scale was selected because it captures core trial-quality domains, including random allocation, allocation concealment, blinding, follow-up, intention-to-treat analysis, and reporting of between-group comparisons and variability estimates. However, we acknowledge that PEDro was originally developed in the context of physiotherapy and rehabilitation research (18) and does not specifically evaluate outcome-domain issues unique to cognitive assessment. Each study was independently evaluated by three reviewers selected from FS-O, VR, NV, PA-H, ST-V, and GJ. The first PEDro item (eligibility criteria) was not included in the total score, resulting in a maximum score of 10. Scores between 6 and 8 were considered indicative of good methodological quality, whereas scores of 9–10 were considered excellent. Disagreements were resolved by discussion with GJ and ST-V. No studies were excluded on the basis of PEDro score. Individual PEDro ratings are presented in Supplementary Table S2.

### 2.8 Statistical analysis

Statistical analyses were performed using Stata version 18 (StataCorp, College Station, TX, USA). Meta-analyses were conducted when at least three studies or independent comparisons reported extractable quantitative data for the same outcome or for outcomes judged sufficiently comparable. “Sufficiently comparable” was defined as data derived from the same instrument or from directly comparable measures assessing the same construct, in clinically compatible populations, with effect directions that could be harmonized. Outcomes were synthesized narratively rather than quantitatively when fewer than three studies or independent comparisons were available, when instruments or constructs were not sufficiently comparable, when numerical data were incomplete or not extractable, or when outcomes were reported only at baseline or in formats unsuitable for pooled effect-size estimation.

For reporting purposes, a unique study was counted only once, irrespective of the number of eligible outcomes or intervention arms. A study–outcome contribution was counted once for each outcome-specific meta-analysis to which a study contributed, whereas an intervention comparison was counted once for each active intervention-versus-control contrast included in the statistical model. Crossover trials were eligible for quantitative synthesis only when an effect estimate and variance accounting for within-participant dependency were available and compatible with the effect measure used in the corresponding meta-analysis. Clinically distinct populations were not combined with existing subgroups when they did not address a comparable clinical context; a separate quantitative subgroup was formed only when the minimum threshold of three studies or independent comparisons was met. Continuous outcomes were pooled using standardized mean differences (Hedges’ g) with 95% confidence intervals (CI), given the variability in outcome measures across studies. MMSE and RBANS outcomes were analyzed separately because they represent distinct cognitive instruments with different scale properties and sensitivities. For handgrip strength, standardized mean differences were used because studies reported values in different units, including kilograms and Newtons. Because this approach standardizes the effect within each study using the study-specific standard deviation, direct unit conversion was not required for the meta-analysis; the direction of effect was harmonized so that positive values consistently favored the intervention group. For multi-arm trials with a shared placebo group, the placebo sample size was divided as evenly as possible across the relevant intervention comparisons, while the original placebo mean and standard deviation were retained. Thus, the shared placebo group was split as 15 and 15 participants for Akhgarjand et al. and as 8 and 9 participants for Lee et al. This approach prevented double counting of control participants while preserving the two distinct active-intervention comparisons.

Random-effects models with restricted maximum likelihood (REML) estimation were applied in all analyses. Statistical heterogeneity was assessed using the I^2^ statistic, with values >50% indicating substantial heterogeneity. When heterogeneity was substantial, leave-one-out sensitivity analyses were performed for the main outcomes when each iteration retained at least three studies or independent comparisons, consistent with the minimum threshold predefined for quantitative synthesis. In addition, targeted *post hoc* study-level sensitivity analyses were conducted to evaluate the influence of potentially influential studies even when their exclusion left only two unique studies. These two-study analyses were treated solely as exploratory stability checks and not as confirmatory meta-analyses. Subgroup analyses were conducted only when the available data met the same minimum threshold for quantitative synthesis and allowed a clinically interpretable comparison. The comparison between the Alzheimer’s disease and broader cognitive-impairment subgroups was not prespecified in the PROSPERO registration and was conducted *post hoc* after the eligible study populations had been identified. It was therefore treated as exploratory and hypothesis-generating rather than confirmatory. Subgroup differences were evaluated using a formal interaction test; statistical significance within one subgroup and non-significance within another were not interpreted as evidence of a subgroup difference in the absence of a statistically significant interaction. For this analysis, studies were classified according to clinical phenotype as Alzheimer’s disease or broader cognitive impairment. Domain-specific analyses were based on the subscale structure of the cognitive instruments when sufficient data were available. Subgroup analyses by intervention type or dose range were considered but were performed only when a sufficient number of studies was available within each subgroup. This condition was not met for probiotic versus synbiotic comparisons or for dose-range categories within the outcome-specific meta-analyses. Publication bias was not formally assessed because fewer than 10 studies were available for each meta-analysis, which is the minimum recommendation for this type of analysis (19).

## 3 Results

### 3.1 Selected studies

The initial search identified 1,420 records: 1,416 from databases and registers and four through citation searching. After removal of 260 duplicate records, 1,160 titles and abstracts were screened. Of these, 153 full-text reports were assessed for eligibility (Figure 1). Following application of the inclusion and exclusion criteria, 129 full-text reports were excluded. Twenty-four studies were included in the qualitative synthesis (Table 1) (20–43), of which 11 unique studies contributed data to at least one quantitative synthesis (Table 2). Because Kobayashi et al. (31) contributed to both the MMSE and RBANS analyses, these studies represented 12 study–outcome contributions. Akhgarjand et al. (21) and Lee et al. (40) each contributed two active intervention comparisons, resulting in 14 intervention comparisons across all meta-analyses. Studies were considered sufficiently comparable when outcomes were derived from the same instrument or from measures assessing the same construct, in clinically compatible populations, with effect directions that could be harmonized.

### 3.2 Quality assessment

Methodological quality was assessed using the PEDro scale across the 24 placebo-controlled trials included in the qualitative synthesis. PEDro scores ranged from 5 to 10, with a mean score of 7.9. Overall, 16 of the 24 studies scored 8 or higher, indicating generally good methodological quality. Random allocation, between-group statistical comparisons, and reporting of point estimates and measures of variability were fulfilled by all studies. Baseline comparability was also frequently satisfied. In contrast, concealed allocation, blinding of therapists and assessors, and intention-to-treat analysis were less consistently reported. Individual PEDro scores are shown in Supplementary Table S2.

### 3.3 Narrative synthesis of the included studies

A total of 24 placebo-controlled clinical trials were included in the qualitative synthesis, of which 20 evaluated cognitive outcomes (20–39) and four evaluated muscle-related outcomes (40–43). Across all included studies, the review encompassed 1,809 participants, with a sample-size-weighted mean age of 69.96 years; 53.96% were women. Two studies did not report sex distribution (23, 40). Most interventions were conducted in Asia (17/24 studies, 71%), particularly in Japan (*n* = 4) and Korea (*n* = 4), followed by Iran (*n* = 3) and China (*n* = 3). Most interventions were probiotic-based, with fewer studies evaluating synbiotics; no FMT trial met the final eligibility criteria. Detailed microbiome-related findings are presented separately in Section 3.4 and Supplementary Table S6. To further characterize intervention-level heterogeneity, studies were categorized according to strain type and reported dose range in Supplementary Table S3. Included interventions comprised *Lactobacillus*-only probiotics, *Bifidobacterium*-only probiotics, mixed multistrain probiotics, and synbiotic formulations. Among studies reporting CFU-based doses, reported doses ranged from 10^6^ CFU/day to ≥10^12^ CFU/day, although several studies reported dose as product weight, concentration, or per-capsule CFU only, preventing reliable conversion to total daily CFU. All interventions were administered orally, with follow-up durations ranging from 4 to 24 weeks (Table 1). The study populations were clinically heterogeneous, ranging from healthy older adults to individuals with AD, MCI, frailty, and sarcopenia. Cognitive outcomes were assessed using a wide range of instruments, including MMSE, RBANS, Montreal Cognitive Assessment (MoCA), Alzheimer’s Disease Assessment Scale – Cognitive Subscale (ADAS-cog), Korean version of the Consortium to Establish a Registry for Alzheimer’s Disease Neuropsychological Assessment Battery (CERAD-K), NIH Toolbox measures, and several domain-specific neuropsychological tests, whereas muscle-related outcomes were primarily assessed using handgrip strength, with additional measures of physical performance and body composition reported in some studies (Table 1). The cognitive instruments used across studies differed in scale range, domain coverage, and expected sensitivity across clinical populations; these characteristics are summarized in Supplementary Table S4.

### 3.4 Microbiome-related outcomes

Ten of the 24 included trials provided post-intervention data on microbial composition, diversity, or targeted bacterial abundance (Supplementary Table S6). Eight trials used broad microbial-community profiling, whereas two used targeted qPCR approaches. Eight trials reported at least one statistically significant microbiological change following the intervention, while Asaoka et al. (22) did not identify a clear overall community-level change and Wang et al. (38) reported non-significant or inconclusive between-group findings. Only one trial—the Sanborn et al. trial (36), as reported in the complementary publication by Aljumaah et al. (44)—assessed fecal recovery of the administered strain at the end of supplementation. However, no trial evaluated persistence after intervention discontinuation or demonstrated durable engraftment. Two trials reported associations between microbiota-related measures and cognitive outcomes, but only the complementary analysis of the Sanborn trial by Aljumaah et al. (44) linked intervention-related microbial changes with cognitive improvement. No included trial examined associations between microbiota changes and muscle-related outcomes.

### 3.5 Meta-analysis

#### 3.5.1 MMSE

Eight trials reported post-intervention MMSE data. Six unique studies met the methodological and clinical-comparability requirements for pooling and contributed seven intervention comparisons (Table 2; Figure 2). Three studies enrolled participants with Alzheimer’s disease (20, 21, 34) and contributed four comparisons because Akhgarjand et al. (21) included two active intervention arms. Three studies enrolled participants with broader cognitive impairment (22, 28, 31) and contributed three comparisons. Ruiz-Gonzalez et al. (35) and Wang et al. (38) were retained in the qualitative synthesis but were not included in the pooled MMSE analysis. Ruiz-Gonzalez et al. used a randomized crossover design. Treating its probiotic and placebo periods as independent groups would have ignored within-participant dependency, whereas incorporating a paired standardized effect into the model used for the parallel-group trials would have required additional assumptions concerning the within-participant correlation and variance standardization. Wang et al. evaluated a short perioperative intervention in older adults undergoing elective surgery. This acute perioperative setting was not considered clinically comparable with the Alzheimer’s disease or broader cognitive-impairment subgroups, and a separate quantitative subgroup could not be formed because it was represented by only one study. In the *post hoc* exploratory subgroup analysis, the pooled estimate within the Alzheimer’s disease subgroup was positive (Hedges’ *g* = 1.46; 95% CI: 1.14–1.79; *I*^2^ = 8.30%) (Figure 2) (20, 21, 34). Within the broader cognitive-impairment subgroup, the pooled estimate was not statistically significant (*g* = 0.49; 95% CI: −0.78 to 1.77; *I*^2^ = 95.91%) (22, 28, 31). When the studies from both subgroups were considered together, the overall pooled estimate was *g* = 1.02 (95% CI: 0.36–1.68), with high heterogeneity (*I*^2^ = 90.84%). The formal test for subgroup differences was not statistically significant (*Q*_b_ (1) = 2.08, *p* = 0.15), providing no evidence that the intervention effect differed between the Alzheimer’s disease and broader cognitive-impairment populations. Accordingly, the subgroup-specific estimates are descriptive and hypothesis-generating and should not be interpreted as demonstrating greater efficacy in either clinical population. Domain-specific MMSE analyses did not show significant pooled effects for orientation or recall (Supplementary Figure S1). Language and visuospatial outcomes were synthesized narratively because only two studies reported comparable data for each domain.

**Figure 2:**
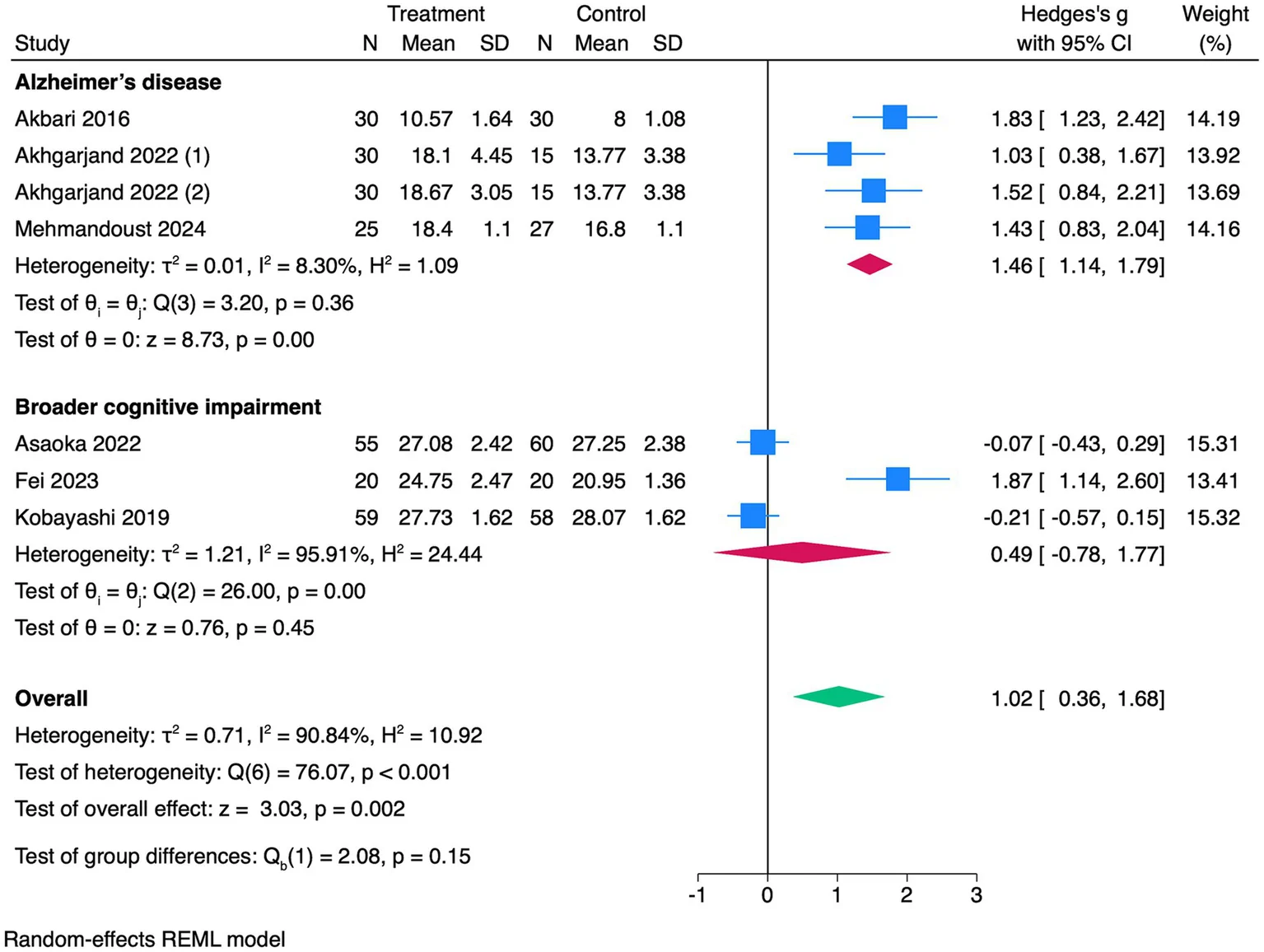
Forest plot of the effects of live microorganism-based interventions on MMSE scores. Six unique studies contributed seven intervention comparisons; Akhgarjand et al. (21) contributed two active intervention arms compared with a shared placebo group. A random-effects meta-analysis evaluated the effects of live microorganism-based interventions on Mini-Mental State Examination (MMSE) scores in aging adults. Studies were grouped according to clinical population (Alzheimer’s disease and broader cognitive impairment). Effect sizes are presented as Hedges’ *g* with 95% confidence intervals (95% CI). Positive effect sizes favor the intervention group over placebo. The pooled analysis showed a positive effect in participants with Alzheimer’s disease (*g* = 1.46; 95% CI: 1.14–1.79; *I*^2^ = 8.30%), whereas no statistically significant pooled effect was observed in participants with broader cognitive impairment (*g* = 0.49; 95% CI: −0.78 to 1.77; *I*^2^ = 95.91%). The overall pooled effect across both subgroups was positive (*g* = 1.02; 95% CI: 0.36–1.68; *I*^2^ = 90.84%; *p* = 0.002). The test for subgroup differences was not statistically significant (*Q*_b_ (1) = 2.08, *p* = 0.15). Squares represent individual study effect sizes, with square size proportional to study weight, and diamonds represent pooled effect estimates.

A targeted *post hoc* study-level sensitivity analysis was conducted for MMSE by excluding Akhgarjand et al. (21) at the study level, meaning that both active intervention arms were removed together. Within the Alzheimer’s disease subgroup, the two remaining studies yielded a positive pooled estimate (Hedges’ *g* = 1.63; 95% CI: 1.21–2.06; *I*^2^ = 0.00%). When all remaining MMSE studies across both clinical subgroups were considered, the overall pooled estimate was *g* = 0.94 (95% CI: 0.03–1.85; *I*^2^ = 94.13%; *p* = 0.043), and the subgroup-interaction test remained non-significant (*Q*_b_ (1) = 2.74, *p* = 0.10). Because the Alzheimer’s disease sensitivity analysis retained only two studies, it was interpreted solely as an exploratory stability check rather than as confirmatory evidence. A conventional leave-one-out analysis was not conducted for the broader cognitive-impairment MMSE subgroup because removing one study would leave only two studies, below the predefined threshold for quantitative synthesis. The MMSE sensitivity results are presented in Supplementary Figures S3, S5.

#### 3.5.2 RBANS

Three unique studies contributed three intervention comparisons to the RBANS quantitative synthesis, including participants with subjective memory complaints and mild cognitive impairment (Table 2). Baseline and final RBANS scores varied markedly across studies and domains, consistent with the clinical and methodological heterogeneity of the included populations. The pooled analysis did not show a significant overall benefit of live microorganism-based interventions (Hedges’ *g* = 0.46; 95% CI: −0.50 to 1.41; *I*^2^ = 93.31%) (Figure 3) (31, 37, 39). Domain-specific meta-analyses likewise did not reveal significant pooled effects for immediate memory, visuospatial/constructional abilities, language, attention, or delayed memory. Although none of these pooled RBANS estimates reached statistical significance, the numerically largest positive trends among RBANS domain-specific estimates were observed for immediate memory, delayed memory, and visuospatial/constructional domains (Supplementary Figure S2). A leave-one-out sensitivity analysis was not conducted because removing one study would leave only two studies, below the predefined threshold for quantitative synthesis. Therefore, the RBANS findings should be interpreted cautiously given the small number of studies and substantial heterogeneity.

**Figure 3:**
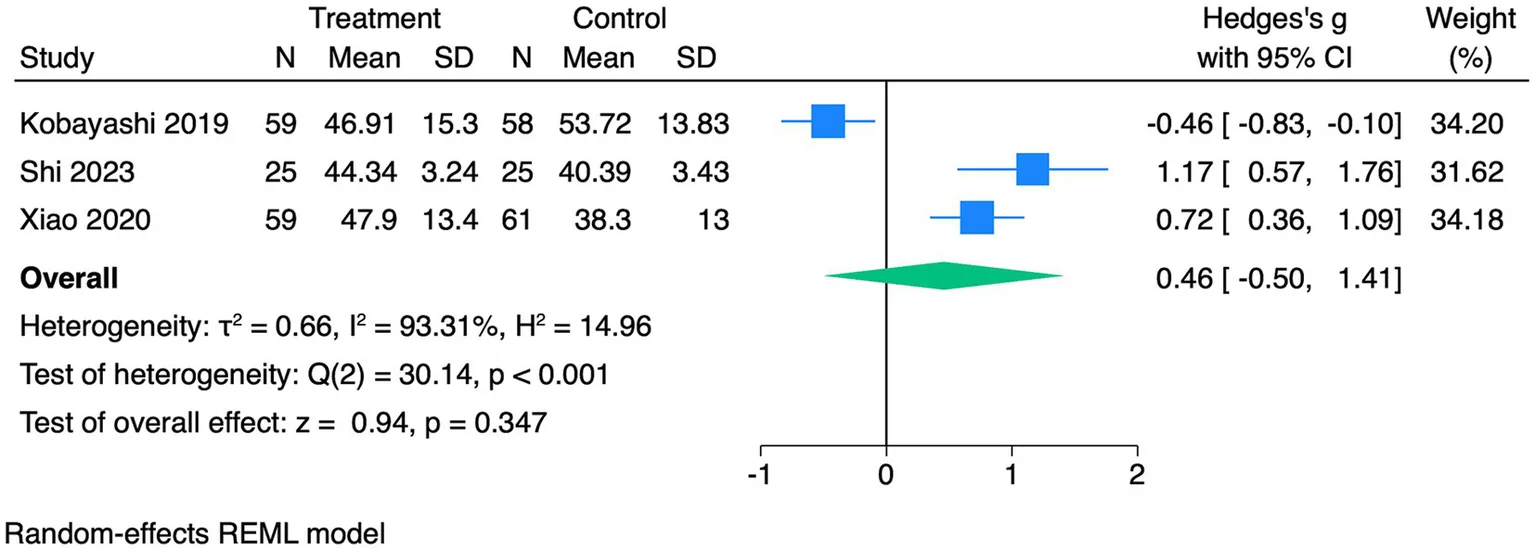
Forest plot of the effects of live microorganism-based interventions on RBANS total score. Three unique studies contributed three intervention comparisons to the RBANS total-score analysis. A random-effects meta-analysis evaluated the effects of live microorganism-based interventions on the RBANS total score in aging adults. Effect sizes are presented as Hedges’ *g* with 95% confidence intervals (95% CI). Positive effect sizes favor the intervention group over placebo. The pooled analysis did not show a statistically significant effect on RBANS total score (*g* = 0.46; 95% CI: −0.50 to 1.41; *I*^2^ = 93.31%). Squares represent individual study effect sizes, with size proportional to study weight, and the diamond represents the pooled effect estimate.

#### 3.5.3 Muscle-related outcomes

Four studies evaluated muscle-related outcomes, of which three unique studies provided sufficiently comparable handgrip-strength data and contributed four intervention comparisons to the meta-analysis (Table 2). Lee et al. (40) contributed two active intervention comparisons against a shared placebo group. These studies included participants with mild frailty, individuals fulfilling at least one frailty criterion, and older men with sarcopenia. Baseline handgrip strength values were generally similar between intervention and placebo groups within each study. At follow-up, the magnitude of improvement appeared greater in the sarcopenia trial than in the frailty-related studies, whereas one study showed limited or no clear advantage for the intervention group. Pooled analysis of handgrip strength showed a modest but statistically significant benefit of live microorganism-based interventions (Hedges’ *g* = 0.46; 95% CI: 0.06–0.85; *I*^2^ = 27.05%; *p* = 0.023) (Figure 4) (40, 42, 43). However, this finding should be interpreted cautiously because the analysis included only three independent studies and four intervention comparisons, two of which were derived from the same study and shared the same placebo group. Therefore, although the I^2^ estimate was low, it is imprecisely estimated with so few studies and does not exclude clinically meaningful between-study differences. Moreover, the magnitude of improvement appeared greater in the sarcopenia trial than in the frailty-related studies, indicating that the pooled estimate may be vulnerable to individual-study influence. A *post hoc* study-level sensitivity analysis was performed after excluding Qaisar et al. (43), whose trial contributed the largest sample. The pooled estimate was attenuated and was no longer statistically significant (Hedges’ *g* = 0.23; 95% CI: −0.27 to 0.73; *I*^2^ = 0.00%; *p* = 0.360). The sensitivity results are presented in Supplementary Figures S4, S5. Because this exclusion left only two unique studies and three intervention comparisons, the analysis was interpreted solely as an exploratory stability check rather than as confirmatory evidence. The attenuation of the pooled estimate indicates that the primary handgrip-strength finding was sensitive to the influence of Qaisar et al. (43).

**Figure 4:**
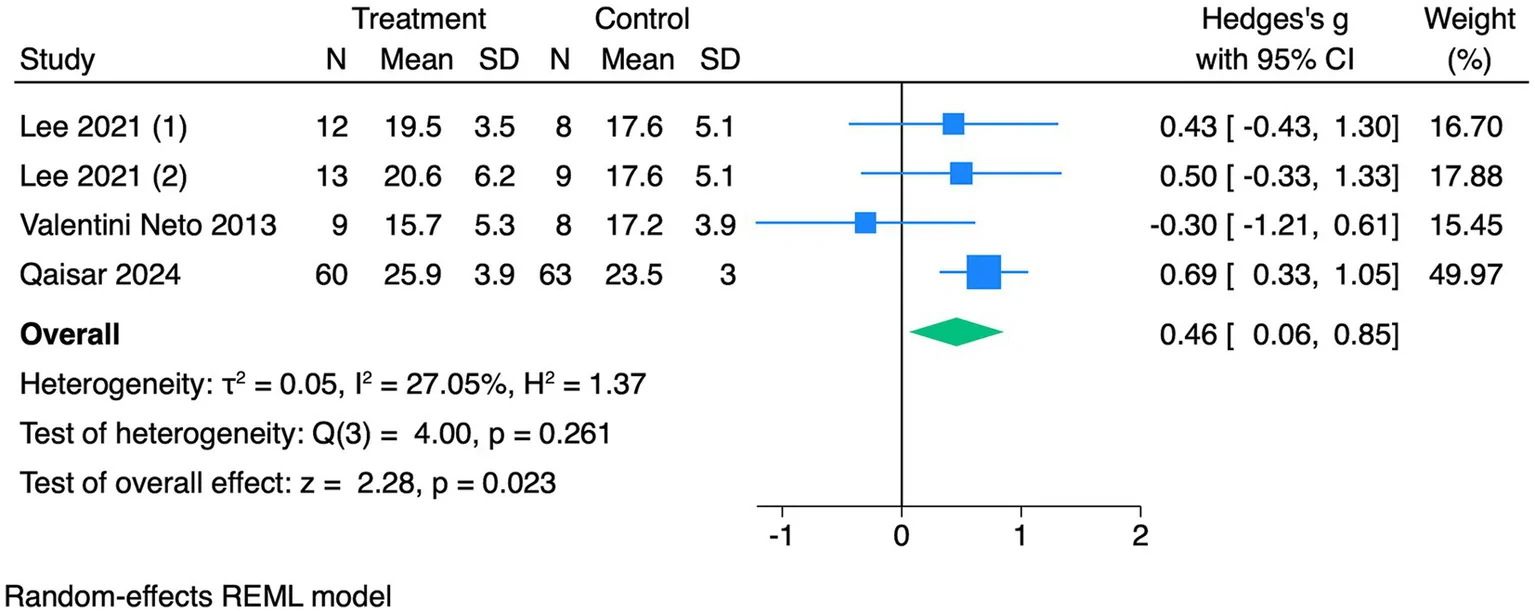
Forest plot of the effects of live microorganism-based interventions on handgrip strength. Three unique studies contributed four intervention comparisons; Lee et al. (40) contributed two active intervention arms compared with a shared placebo group. A random-effects meta-analysis evaluated the effects of live microorganism-based interventions on handgrip strength (HGS) in aging adults. Effect sizes are presented as Hedges’ *g* with 95% confidence intervals (95% CI). Positive effect sizes favor the intervention group over placebo. The pooled analysis showed a statistically significant positive effect on handgrip strength (*g* = 0.46; 95% CI: 0.06–0.85; *I*^2^ = 27.05%; *p* = 0.023). However, because only three independent studies were included, the heterogeneity estimate should be interpreted cautiously. Squares represent individual study effect sizes, with square size proportional to study weight, and the diamond represents the pooled effect estimate.

## 4 Discussion

This systematic review and meta-analysis evaluated whether live microorganism-based interventions influence cognitive and muscle-related outcomes in aging adults. Three findings summarize the available evidence. First, the overall MMSE analysis yielded a positive pooled estimate. In the *post hoc* subgroup analysis, the within-subgroup estimate was positive in participants with Alzheimer’s disease but not statistically significant in those with broader cognitive impairment; however, the interaction test was non-significant and therefore provided no evidence that the intervention effect differed between these populations. Second, RBANS total and domain-specific analyses were not statistically significant and showed substantial heterogeneity. Third, handgrip strength showed a modest positive primary estimate, but this finding was based on only three independent studies and became non-significant after exclusion of Qaisar et al. (43). Collectively, these results identify preliminary short-term signals rather than confirmatory evidence, and they do not establish a clinical phenotype with a greater response to live microorganism-based interventions.

The overall MMSE finding is directionally consistent with previous syntheses, although differences in populations and methods limit direct comparison. Mo et al. (13), in a meta-analysis restricted to adults with MCI or Alzheimer’s disease, reported improvements in global cognition and selected domains. Calzada-Gonzales et al. (12), using a broader adult population, identified possible MMSE and MoCA benefits but rated the certainty of evidence as low to very low. A more recent AD-specific review of double-blind trials also reported a cognitive benefit, accompanied by substantial heterogeneity (45). The present review adds an aging-focused perspective, but it cannot determine whether clinical phenotype explains variation in response. The Alzheimer’s disease versus broader cognitive-impairment comparison was not prespecified in PROSPERO, and its formal interaction test was non-significant (*Q*_b_ (1) = 2.08, *p* = 0.15). Moreover, a targeted study-level sensitivity analysis excluding both intervention arms from Akhgarjand et al. (21) retained only two Alzheimer’s disease studies. Although the estimate remained positive (*g* = 1.63; 95% CI: 1.21–2.06), the subgroup-interaction test also remained non-significant (*Q*_b_ (1) = 2.74, *p* = 0.10). This analysis therefore serves only as an exploratory stability check and does not demonstrate an Alzheimer’s disease-specific treatment effect.

Two additional considerations further constrain interpretation of MMSE. First, a separate subgroup for the two studies that did not contribute to the pooled MMSE analysis was not appropriate because they represented distinct settings: one was a crossover trial in healthy older adults and the other evaluated an acute perioperative intervention. Second, the included interventions lasted only 4–24 weeks. Any MMSE improvement should therefore be interpreted as a short-term change in cognitive performance or symptomatic/functional status, rather than evidence that these interventions modify neurodegeneration, alter disease trajectory, or produce durable clinical benefit.

The null RBANS findings should not be interpreted as disproving microbiota-related cognitive effects; instead, they illustrate the fragmentation of the evidence. Only three studies contributed RBANS data, and they enrolled healthy aging adults, individuals with subjective memory complaints, and participants with MCI. These populations differ in baseline cognitive reserve and degree of impairment and may also differ in metabolic and inflammatory status, gut microbial composition, and capacity to respond to supplementation. In addition, RBANS evaluates more specific neuropsychological domains than MMSE and may be more sensitive to methodological differences among trials. Immediate memory, delayed memory, and visuospatial/constructional scores showed the largest numerical trends, broadly consistent with earlier domain-focused syntheses (13, 46), but none reached statistical significance in the present dataset. Both the RBANS analysis and the broader cognitive-impairment MMSE subgroup were therefore underpowered and heterogeneous. Leave-one-out analyses were not undertaken because each exclusion would have left only two studies, below the predefined threshold for quantitative synthesis.

A central contribution of this review is its functional-aging framework. Earlier syntheses generally examined cognition or sarcopenia separately, frequently combined broader adult age ranges, and sometimes grouped probiotics with non-living or indirectly microbiota-modulating strategies. By restricting eligibility to aging adult populations and to live microorganism-based interventions, and by evaluating cognitive and muscle-related outcomes together, the present review addresses two complementary domains of functional ability. This narrower scope avoids conflating probiotics and synbiotics with interventions such as isolated fibers, polyphenols, exercise, or postbiotics, while still recognizing the clinical interdependence of cognitive function, muscle strength, autonomy, and resilience in later life (12–14).

Within this framework, the handgrip-strength finding is relevant but not definitive. The primary pooled estimate was positive and estimated heterogeneity was relatively low, yet only three independent studies were available. After excluding the larger trial by Qaisar et al. (43), the estimate was attenuated and no longer statistically significant (*g* = 0.23; 95% CI: −0.27 to 0.73), with only two unique studies and three comparisons remaining. Thus, the sensitivity analysis is exploratory and shows that the primary finding is susceptible to individual-study influence. The direction of the estimate agrees with Besora-Moreno et al. (14), who reported improved handgrip strength and possible gait-speed benefits, although that review combined probiotics, prebiotics, and synbiotics and used mean differences, precluding direct numerical comparison. Biological plausibility is supported by the gut-muscle literature, which implicates inflammatory regulation, intestinal barrier integrity, short-chain fatty acids, nutrient and amino acid availability, anabolic-catabolic signaling, and interactions with myokines and neuroendocrine pathways (4, 47). Nevertheless, the present evidence cannot establish handgrip strength as a particularly responsive endpoint for probiotics or synbiotics. Handgrip remains a standardized and clinically relevant component of muscle function, but it does not capture daily functioning, fall risk, quality of life, or healthy aging as a whole. These broader functional outcomes were reported too inconsistently for quantitative synthesis.

Gut-brain and gut-muscle pathways also provide a plausible biological context for the cognitive and muscle findings. Microbial metabolites, immune and neuroendocrine signaling, vagal pathways, intestinal and blood–brain barrier integrity, and neuroinflammation may influence brain aging and neurodegeneration (2, 3), while related inflammatory and metabolic pathways may affect skeletal muscle (4). However, the review did not directly test these mechanisms, identify causal strains or metabolites, or demonstrate that mechanistic pathways differ between Alzheimer’s disease and broader cognitive impairment.

The microbiological findings reinforce this caution. Only 10 of the 24 included trials reported post-intervention data on microbial composition, diversity, or targeted bacterial abundance, and the analytical methods and reported outcomes varied substantially (Supplementary Table S6). Eight trials reported at least one significant microbiological change, but only two examined associations between microbiota-related measures and cognitive outcomes. Moreover, only the complementary analysis of the Sanborn trial by Aljumaah et al. (44) linked an intervention-related microbial change with cognitive improvement. No included trial related microbiota changes to muscle outcomes. That complementary analysis was also the only one to assess fecal recovery of an administered strain at the end of supplementation; no study evaluated persistence after intervention discontinuation or demonstrated durable engraftment. Consequently, the observed clinical signals cannot yet be firmly attributed to specific microbiome changes, particular microbial strains, or sustained colonization. Future mechanistic trials will need longitudinal microbiome sampling and integrated inflammatory, metabolic, and clinical measurements to distinguish transient exposure from biologically meaningful ecosystem change.

FMT remains a conceptually relevant but currently unrepresented intervention category in the eligible evidence. It was included in the search framework because it transfers a complex living microbial community and may reshape gut ecology more extensively than a defined probiotic or synbiotic formulation (10). Clinical studies have examined cognitive outcomes following FMT (48), but no published FMT randomized controlled trial met the final eligibility criteria of this review. Bajaj et al. (49) evaluated capsular FMT in patients with cirrhosis and recurrent hepatic encephalopathy who were receiving lactulose and rifaximin; the target condition, co-interventions, and disease-specific cognitive assessment were not aligned with aging-related cognitive decline or muscle function. FMT trials in neurological diseases such as Parkinson’s disease were likewise designed around disease-specific aims and outcomes rather than functional aging (50). The present evidence base therefore derives exclusively from probiotics and synbiotics. This absence should not be interpreted as evidence against FMT, but as an evidence gap. The ARMOR protocol (51), which evaluates oral FMT from young physically active donors in community-dwelling adults aged 65–84 years using cognitive, physical, metabolic, and shotgun metagenomic outcomes, illustrates the type of trial that may address this gap. Because it is currently a protocol without outcome data, it was not eligible for inclusion, but future updates should reassess FMT as results from this and similar trials become available.

The review has several methodological strengths. It focused on placebo-controlled trials in aging adult populations, applied a coherent intervention definition, and separated qualitative from quantitative synthesis so that clinically or methodologically incompatible outcomes were not pooled. Joint consideration of cognition and muscle-related function also provides a broader functional-aging perspective than reviews restricted to one domain. Methodological quality was generally good according to PEDro, and description of studies by clinical phenotype helped make the substantial population heterogeneity transparent. Nevertheless, concealed allocation, blinding of therapists or assessors, and intention-to-treat procedures were not consistently reported. These design choices strengthen interpretability, but they do not overcome limitations in the reporting and size of the underlying evidence.

The most important limitation is the small quantitative evidence base. The MMSE subgroup comparison was *post hoc*, the interaction tests were non-significant, and targeted sensitivities retaining only two independent studies cannot support confirmatory inference. RBANS, the broader cognitive-impairment MMSE subgroup, and handgrip strength were each informed by few studies, limiting precision and increasing susceptibility to study-level effects. Publication bias could not be assessed because each meta-analysis included fewer than 10 studies, and meta-regression would have been underpowered, unstable, and at risk of spurious findings (52). Accordingly, heterogeneity in the cognitive analyses remains largely unexplained.

Clinical and methodological heterogeneity provide additional limitations. Strains, doses, combinations, delivery matrices, duration, and prebiotic components varied markedly (Supplementary Table S3), and the small outcome-specific datasets precluded reliable comparisons by intervention type, strain category, or dose. In particular, synbiotics contributed only one study to the quantitative MMSE analysis and one to handgrip strength, while RBANS was represented only by *Bifidobacterium*-based interventions. The present evidence therefore cannot determine whether particular genera, species, strains, doses, or synbiotic formulations are superior. Follow-up was short, most trials were conducted in Asia, and English-language eligibility may have introduced regional and language bias. Geographic differences in habitual diet, fiber and fermented-food intake, and baseline microbiome composition may also affect colonization resistance and metabolic or inflammatory responses, limiting generalizability to Western and underrepresented South Asian populations. Differences among cognitive instruments in construct, scale, domain coverage, sensitivity, and ceiling or floor effects further complicate interpretation; these considerations are summarized in Supplementary Table S4. Sex-stratified analyses were not possible because two studies did not report sex distribution (23, 40) and most trials did not provide outcomes by sex. Finally, although PEDro captures important RCT features, it was developed for physiotherapy and rehabilitation research (18) and may not fully address issues specific to neuropsychological assessment, including learning effects, test sensitivity, domain coverage, and the clinical relevance of score changes.

The clinical implications should therefore remain cautious. Current evidence does not justify routine use of live microorganism-based interventions as standard therapy for cognitive decline or sarcopenia, nor does it support prioritizing Alzheimer’s disease or another cognitive phenotype for treatment. Probiotics and synbiotics remain relevant candidates for further investigation in populations characterized by frailty, inflammation, neurodegeneration, or microbiota disruption; however, the current evidence is insufficient to support their adjunctive clinical use. The review thus identifies both a plausible functional-aging research direction and the methodological conditions required for progress. Future trials should prespecify clinical subgroup hypotheses, be powered for treatment-by-subgroup interactions, and distinguish AD, MCI, subjective cognitive decline, and healthy aging. Longer follow-up and harmonized outcome sets are needed to separate transient symptomatic changes from durable functional benefit. Muscle studies should combine handgrip strength with physical-performance tests, falls, ADL/IADL, quality of life, and body-composition measures. Across both domains, standardized strain-level reporting, baseline diet and microbiome characterization, assessment of post-intervention persistence, inflammatory and metabolic biomarkers, adherence measures, geographically diverse recruitment, and sex-disaggregated outcomes will be essential to determine which interventions work, for whom, through which mechanisms, and for how long.

## 5 Conclusion

The present systematic review and meta-analysis identified preliminary short-term functional signals in aging adults, including positive primary pooled estimates for overall MMSE and handgrip strength. However, the handgrip-strength estimate was attenuated and was no longer statistically significant after exclusion of Qaisar et al., indicating sensitivity to individual-study influence. Overall, the quantitative evidence base remains small and heterogeneous. The *post hoc* exploratory comparison between participants with Alzheimer’s disease and those with broader cognitive impairment did not yield a statistically significant subgroup interaction; therefore, no differential effect between these clinical populations can be inferred. The available evidence was derived from probiotic and synbiotic trials, as no published FMT randomized controlled trial met the final eligibility criteria. Larger and longer randomized trials with standardized outcomes and prospectively specified subgroup hypotheses are required before clinical or population-specific recommendations can be made.

## Data Availability

The data supporting the conclusions of this article will be made available by the corresponding authors, upon request.
