# Supplementary material for: Can live microbial interventions support healthy aging? A systematic review and meta-analysis of cognitive and muscle outcomes in aging adults
Source: Front Nutr. 2026 Sep 16;13:1869929. doi: 10.3389/fnut.2026.1869929 (PMC13623691; doi:10.3389/fnut.2026.1869929)
Supplement: Supplementary file 1 [file Table_1.DOCX]

**SUPPLEMENTARY MATERIAL**

**Full electronic search strategies by database**

Search date: 7 May 2025. Manual citation-search update: December 2025. The strategies combined controlled vocabulary terms, where available, with free-text terms across intervention, aging, and cognitive or muscle-related outcome domains.

| **Database** | **Search platform** | **Main search domains** | **Boolean structure** | **Filters and limits** | **Records retrieved** |
| --- | --- | --- | --- | --- | --- |
| Embase | Ovid | Live microorganism-based interventions; aging-related terms; cognitive and muscle-related outcomes | (microbiota OR fecal microbiota transplantation OR probiotics) AND (aging OR longevity OR elderly OR healthy aging) AND (cognitive outcomes OR muscle-related outcomes) | Human studies; English language; adult/aged population; reviews excluded | 488 |
| PubMed/MEDLINE | PubMed | MeSH and Title/Abstract terms for microbiota, probiotics, fecal microbiota transplantation, aging, cognition, frailty, sarcopenia, and skeletal muscle | (intervention terms) AND (aging terms) AND (cognitive OR muscle-related outcome terms) | Randomized Controlled Trial; Humans; English language | 68 |
| Cochrane CENTRAL | CENTRAL | Free-text terms for microbiota-based interventions, aging, cognition, frailty, sarcopenia, and skeletal muscle | (intervention terms) AND (aging terms) AND (outcome terms) | None | 686 |
| Web of Science | Core Collection | Topic search terms for microbiota-based interventions, aging, cognitive outcomes, frailty, sarcopenia, skeletal muscle, and clinical trial design | TS=(intervention terms) AND TS=(aging terms) AND TS=(outcome terms) AND TS=(clinical trial terms) | None beyond topic search terms | 174 |
| Additional search methods | Manual citation searching | Reference lists of eligible studies | Citation searching | Not applicable | 4 |

**Database-specific full search strategies**

**Embase via Ovid**

| **Search block** | **Search terms and combinations** |
| --- | --- |
| Intervention | microflora/ OR microbiome/ OR intestine flora/ OR microbiota.tw. OR microbiome.tw. OR fecal microbiota transplantation/ OR fecal microbiota transplantation.tw. OR probiotic agent/ OR probiotic.tw. OR probiotics.tw. |
| Aging | aging/ OR aged/ OR aging.tw. OR ageing.tw. OR aged.tw. OR longevity/ OR longevity.tw. OR elderly.tw. OR life extension/ OR life extension.tw. OR healthy aging/ OR healthy aging.tw. OR healthy ageing.tw. |
| Outcomes: cognitive | cognition/ OR cognition.tw. OR cognitive aging/ OR cognitive aging.tw. OR mild cognitive impairment/ OR mild cognitive impairment.tw. OR dementia/ OR dementia.tw. OR neuroprotection/ OR neuroprotection.tw. |
| Outcomes: muscle-related | frailty/ OR frailty.tw. OR skeletal muscle/ OR skeletal muscle.tw. OR muscle tissue/ OR muscle tissue.tw. OR sarcopenia/ OR sarcopenia.tw. |
| Final combination | (intervention block) AND (aging block) AND (cognitive outcomes OR muscle-related outcomes) |
| Limits | Human studies; English language; adult/aged population; reviews excluded |

**PubMed/MEDLINE**

| **Search block** | **Search terms and combinations** |
| --- | --- |
| Intervention | "Microflora"[MeSH] OR "Microbiota"[MeSH] OR "Microbiome"[MeSH] OR "Fecal Microbiota Transplantation"[MeSH] OR "Intestine Flora"[MeSH] OR "Probiotics"[MeSH] OR "Probiotic"[MeSH] OR microbiota[Title/Abstract] OR microbiome[Title/Abstract] OR gut microbiota[Title/Abstract] OR probiotics[Title/Abstract] OR "fecal microbiota transplantation"[Title/Abstract] |
| Aging | "Aging"[MeSH] OR "Aged"[MeSH] OR "Aged, 80 and over"[MeSH] OR "Life Extension"[MeSH] OR "Healthy Aging"[MeSH] OR aging[Title/Abstract] OR ageing[Title/Abstract] OR aged[Title/Abstract] OR elderly[Title/Abstract] OR longevity[Title/Abstract] OR "healthy aging"[Title/Abstract] OR "healthy ageing"[Title/Abstract] |
| Outcomes | "Cognition"[MeSH] OR "Cognitive Aging"[MeSH] OR "Mild Cognitive Impairment"[MeSH] OR "Dementia"[MeSH] OR "Neuroprotection"[MeSH] OR "Frailty"[MeSH] OR "Sarcopenia"[MeSH] OR "Skeletal Muscle"[MeSH] OR "Muscle Tissue"[MeSH] OR cognition[Title/Abstract] OR "cognitive aging"[Title/Abstract] OR "mild cognitive impairment"[Title/Abstract] OR dementia[Title/Abstract] OR neuroprotection[Title/Abstract] OR frailty[Title/Abstract] OR sarcopenia[Title/Abstract] OR "skeletal muscle"[Title/Abstract] OR "muscle tissue"[Title/Abstract] |
| Final combination | (intervention block) AND (aging block) AND (outcome block) |
| Filters | Randomized Controlled Trial; Humans; English language |

**Cochrane CENTRAL**

| **Search block** | **Search terms and combinations** |
| --- | --- |
| Intervention | microbiota OR microbiome OR "gut microbiota" OR probiotics OR "fecal microbiota transplantation" |
| Aging | aging OR ageing OR aged OR elderly OR longevity OR "healthy aging" OR "healthy ageing" |
| Outcomes | cognition OR "cognitive aging" OR "mild cognitive impairment" OR dementia OR neuroprotection OR frailty OR sarcopenia OR "skeletal muscle" OR "muscle tissue" |
| Final combination | (intervention block) AND (aging block) AND (outcome block) |
| Filters | None |

**Web of Science**

| **Search block** | **Search terms and combinations** |
| --- | --- |
| Intervention | TS=(microbiota OR microbiome OR "intestinal flora" OR "fecal microbiota transplantation" OR probiotics) |
| Aging | TS=(aging OR ageing OR aged OR elderly OR "life extension" OR "healthy aging" OR "healthy ageing" OR longevity) |
| Outcomes | TS=(cognition OR "cognitive aging" OR "mild cognitive impairment" OR MCI OR dementia OR neuroprotection OR frailty OR "skeletal muscle" OR "muscle tissue" OR sarcopenia) |
| Study design | TS=("clinical trial" OR "intervention study" OR RCT OR "randomized controlled trial") |
| Final combination | (intervention block) AND (aging block) AND (outcome block) AND (study design block) |
| Filters | None beyond topic search terms |

**Additional search methods**

| **Search block** | **Search terms and combinations** |
| --- | --- |
| Manual citation searching | Reference lists of eligible studies were manually screened to identify additional relevant publications through citation searching. This process included a targeted check for potentially eligible synbiotic interventions. Four additional records were identified through citation searching. |

Abbreviations: CENTRAL, Cochrane Central Register of Controlled Trials; FMT, fecal microbiota transplantation; MeSH, Medical Subject Headings; RCT, randomized controlled trial; TS, topic search; tw, text word.

**Supplementary Table S1. Full search strategies for all databases.** This table presents the complete search strategies used for each electronic database, including Embase (via Ovid), PubMed/MEDLINE, Cochrane Central Register of Controlled Trials (CENTRAL), and Web of Science. The electronic database search was conducted on 7 May 2025, and a manual citation-search update was performed in December 2025. The strategies combined controlled vocabulary terms (MeSH and Emtree) and free-text terms related to microbiota-based interventions, aging, and cognitive or muscle-related outcomes. Database-specific syntax and filters were applied, including restrictions to human studies, English-language publications, and randomized controlled trials when available. The number of records retrieved from each database is also reported.

| Study | 1 | 2 | 3 | 4 | 5 | 6 | 7 | 8 | 9 | 10 | 11 | Score |
| --- | --- | --- | --- | --- | --- | --- | --- | --- | --- | --- | --- | --- |
| *Studies evaluating cognitive function* | | | | | | | | | | | | |
| Akbari et al. (2016) | - | Yes | Yes | Yes | Yes | No | Yes | Yes | Yes | Yes | Yes | 9 |
| Akhgarjand et al. (2022) | - | Yes | Yes | Yes | Yes | Yes | Yes | Yes | Yes | Yes | Yes | 10 |
| Asaoka et al. (2022) | - | Yes | Yes | Yes | Yes | Yes | Yes | Yes | Yes | Yes | Yes | 10 |
| Azuma et al. (2023) |  | Yes | No | Yes | Yes | Yes | Yes | No | No | Yes | Yes | 7 |
| Baek et al. (2025) | - | Yes | No | Yes | Yes | No | No | Yes | Yes | Yes | Yes | 7 |
| Bartos et al. (2023) | - | Yes | Yes | Yes | Yes | Yes | Yes | Yes | No | Yes | Yes | 9 |
| Chung et al. (2014) | - | Yes | No | Yes | Yes | Yes | Yes | Yes | No | Yes | Yes | 8 |
| Eastwood et al. (2025) |  | Yes | No | Yes | Yes | Yes | Yes | Yes | Yes | Yes | Yes | 9 |
| Fei et al. (2023) | - | Yes | No | Yes | No | No | No | Yes | No | Yes | Yes | 5 |
| Hwang et al. (2019) | - | Yes | Yes | Yes | Yes | Yes | Yes | Yes | Yes | Yes | Yes | 10 |
| Kim et al. (2021) | - | Yes | Yes | Yes | Yes | Yes | No | No | No | Yes | Yes | 7 |
| Kobayashi et al. (2019) | - | Yes | Yes | No | Yes | No | Yes | Yes | Yes | Yes | Yes | 8 |
| Lazou-Ahrén et al. (2025) | - | Yes | Yes | Yes | Yes | Yes | No | Yes | Yes | Yes | Yes | 9 |
| Louzada and Ribeiro. (2020) | - | Yes | Yes | Yes | Yes | Yes | No | No | Yes | Yes | Yes | 8 |
| Mehmandoust et al. (2024) | - | Yes | Yes | Yes | Yes | Yes | No | Yes | No | Yes | Yes | 8 |
| Ruiz-Gonzalez et al. (2025) | - | Yes | Yes | No | Yes | Yes | Yes | No | Yes | Yes | Yes | 8 |
| Sanborn et al. (2020) | - | Yes | No | Yes | Yes | Yes | No | No | No | Yes | Yes | 6 |
| Shi et al. (2023) | - | Yes | Yes | Yes | Yes | Yes | Yes | No | No | Yes | Yes | 8 |
| Wang et al. (2021) | - | Yes | Yes | Yes | Yes | Yes | Yes | Yes | No | Yes | Yes | 9 |
| Xiao et al. (2020) | - | Yes | Yes | Yes | Yes | No | No | Yes | Yes | Yes | Yes | 8 |
| *Studies evaluating muscular function* | | | | | | | | | | | | |
| Lee et al. (2021) | - | Yes | No | Yes | Yes | Yes | No | No | No | Yes | Yes | 6 |
| Lee et al. (2025) | - | Yes | No | Yes | Yes | No | No | No | No | Yes | Yes | 5 |
| Neto et al. (2013) | - | Yes | Yes | Yes | Yes | Yes | No | Yes | No | Yes | Yes | 8 |
| Qaisar et al. (2024) | - | Yes | No | Yes | Yes | Yes | No | Yes | No | Yes | Yes | 7 |

**Supplementary Table S2. PEDro assessment of all 24 placebo-controlled randomized trials included in the qualitative synthesis.** The PEDro score was calculated as the sum of items 2–11 (range 0–10). The numbers in the column headers correspond to the PEDro scale criteria. Item 1 (eligibility criteria) was not included in the total score as it relates to external validity. Each satisfied criterion (“Yes”) was awarded 1 point. Criteria: (2) random allocation; (3) concealed allocation; (4) baseline comparability; (5) blinding of subjects; (6) blinding of therapists; (7) blinding of assessors; (8) adequate follow-up (>85% of participants); (9) intention-to-treat analysis; (10) between-group statistical comparisons; and (11) point estimates and measures of variability reported.

**Summary of intervention categories**

| **Intervention classification** | **Number of studies** |
| --- | --- |
| Lactobacillus-only probiotic | 7 |
| Bifidobacterium-only probiotic | 5 |
| Mixed multistrain probiotic | 6 |
| Synbiotic | 5 |
| Separate single-strain probiotic arms | 1 |

**Detailed classification**

| **Study** | **Outcome domain** | **Meta-analysis contribution** | **Reviewer-requested classification** | **Microbial composition / formulation** | **Reported dose** | **Dose range category** | **Delivery matrix** | **Duration** |
| --- | --- | --- | --- | --- | --- | --- | --- | --- |
| Akbari et al. (2016) | Cognitive | MMSE | Mixed multistrain probiotic | Four-strain probiotic*: Lactobacillus acidophilus, L. casei, Bifidobacterium bifidum, and L. fermentum* | 2 x 10^9^ CFU/g for each strain; daily CFU not derivable from reported concentration | Not convertible | Probiotic milk (200 mL/day) | 12 weeks |
| Akhgarjand et al. (2022) | Cognitive | MMSE | Separate single-strain probiotic arms | Arm 1: *Lactobacillus rhamnosus* HA-114; Arm 2: *Bifidobacterium longum* R0175 | 1 x 10^15^ CFU twice daily for each active arm | >=10^12^ CFU/day | Capsules | 12 weeks |
| Asaoka et al. (2022) | Cognitive | MMSE | Bifidobacterium-only probiotic | *Bifidobacterium* breve MCC1274 | 2 x 10^10^ CFU/day | 10^10^-<10^12^ CFU/day | Sachet; maize-starch placebo | 24 weeks |
| Azuma et al. (2023) | Cognitive | Qualitative only | Synbiotic | *Bifidobacterium animalis* subsp. lactis GCL2505 + inulin | 1 x 10^10^ CFU/day + inulin 2 g/day | 10^10^-<10^12^ CFU/day | Dairy drink | 12 weeks |
| Baek et al. (2025) | Cognitive | Qualitative only | Lactobacillus-only probiotic | *Lactobacillus delbrueckii* subsp. lactis CKDB001 | 5 x 10^9^ CFU/day | 10^8^-<10^10^ CFU/day | Capsules | 12 weeks |
| Bartos et al. (2023) | Cognitive | Qualitative only | Synbiotic | *Streptococcus thermophilus GH, S. salivarius GH NEXARS, Lactobacillus plantarum GH, Pediococcus pentosaceus GH* + fiber tablets | 10^6^ CFU/day + fiber tablets | 10^6^-<10^8^ CFU/day | Probiotic tablet plus fiber tablets | 3 months per phase |
| Chung et al. (2014) | Cognitive | Qualitative only | Lactobacillus-only probiotic | *Lactobacillus helveticus* IDCC3801 fermented milk | 125, 250, or 500 mg LHFM; CFU not reported | Not reported as CFU | Fermented milk tablets | 12 weeks |
| Eastwood et al. (2025) | Cognitive | Qualitative only | Mixed multistrain probiotic | Nine strains across Bifidobacterium, Lactobacillus-related genera, and Lactococcus | 5 x 10^9^ CFU/day | 10^8^-<10^10^ CFU/day | Sachet with maize starch, maltodextrin, vegetable protein, and potassium chloride | 8 weeks |
| Fei et al. (2023) | Cognitive | MMSE | Mixed multistrain probiotic | Eighteen-strain formulation including Lactobacillus-related genera, Lactococcus, and Bifidobacterium | >2 x 10^10^ CFU/g; 2 g/day (>4 x 10^10^ CFU/day) | 10^10^-<10^12^ CFU/day | Capsule; starch placebo | 12 weeks |
| Hwang et al. (2019) | Cognitive | Qualitative only | Lactobacillus-only probiotic | *Lactobacillus plantarum* C29-fermented soybean (DW2009) | 800 mg/day; CFU not reported | Not reported as CFU | Capsules; fermented soybean product | 12 weeks |
| Kim et al. (2021) | Cognitive | Qualitative only | Bifidobacterium-only probiotic | *Bifidobacterium bifidum* BGN4 and *Bifidobacterium longum* BORI | 1 x 10^9^ CFU as reported; daily total unclear | 10^8^-<10^10^ CFU/day* | Capsules in soybean oil | 12 weeks |
| Kobayashi et al. (2019) | Cognitive | MMSE; RBANS | Bifidobacterium-only probiotic | *Bifidobacterium breve* A1 | >2 x 10^10^ CFU/day | 10^10^-<10^12^ CFU/day | Capsules; corn starch placebo | 12 weeks |
| Lazou-Ahrén et al. (2025) | Cognitive | Qualitative only | Lactobacillus-only probiotic | *Lactobacillus plantarum* HEAL9; one arm combined with freeze-dried berries | CFU not reported in extracted table | Not reported as CFU | Powder/sachet; sour milk or breakfast flakes as vehicle | 12 weeks |
| Louzada and Ribeiro (2020) | Cognitive | Qualitative only | Synbiotic | FOS + *Lactobacillus paracasei, L. rhamnosus, L. acidophilus, and Bifidobacterium lactis* | 10^8^-10^9^ CFU plus FOS 6 g; 12 g/day product | 10^8^-<10^10^ CFU/day* | Powder/sachets; maltodextrin placebo | 24 weeks |
| Mehmandoust et al. (2024) | Cognitive | MMSE | Synbiotic | Seven-strain formulation including *Lactobacillus spp., Bifidobacterium spp., Streptococcus thermophilus* + FOS | 10^9^ CFU + FOS | 10^8^-<10^10^ CFU/day | Capsules; maltodextrin placebo | 12 weeks |
| Ruiz-Gonzalez et al. (2025) | Cognitive | Qualitative only- MMSE reported but not pooled | Mixed multistrain probiotic | *Lactobacillus rhamnosus* and *Bifidobacterium lactis* | 3.3 x 10^9^ CFU/day | 10^8^-<10^10^ CFU/day | Capsules; potato starch placebo | 10 weeks |
| Sanborn et al. (2020) | Cognitive | Qualitative only | Lactobacillus-only probiotic | *Lactobacillus rhamnosus* GG | 2 x 10^10^ CFU/day | 10^10^-<10^12^ CFU/day | Capsules; microcrystalline cellulose placebo | 3 months |
| Shi et al. (2023) | Cognitive | RBANS | Bifidobacterium-only probiotic | *Bifidobacterium longum* BB68S | 5 x 10^10^ CFU/day | 10^10^-<10^12^ CFU/day | Sachet; maltodextrin placebo | 8 weeks |
| Wang et al. (2021) | Cognitive | Qualitative only-MMSE reported but not pooled | Mixed multistrain probiotic | *Bifidobacterium longum, Lactobacillus acidophilus*, and *Enterococcus faecalis* | >1 x 10^7^ CFU/capsule for each species; daily total unclear | Not fully convertible | Capsules; lactose placebo | 8 weeks |
| Xiao et al. (2020) | Cognitive | RBANS | Bifidobacterium-only probiotic | *Bifidobacterium breve* A1 | >1 x 10^10^ CFU/day | 10^10^-<10^12^ CFU/day | Capsules; maize starch placebo | 16 weeks |
| Lee et al. (2021) | Muscle | Handgrip strength | Lactobacillus-only probiotic | *Lactobacillus plantarum* TWK10 | 2 x 10^10^ or 6 x 10^10^ CFU/day | 10^10^-<10^12^ CFU/day | Capsules; maltodextrin/microcrystalline cellulose placebo | 18 weeks |
| Lee et al. (2025) | Muscle | Qualitative only | Lactobacillus-only probiotic | Live *Lactobacillus paracasei* PS23; heat-treated comparator arm also included | 2 x 10^10^ CFU/day for live arm; 2 x 10^10^ cells/day for heat-treated arm | 10^10^-<10^12^ CFU/day | Capsules; microcrystalline cellulose placebo | 12 weeks |
| Neto et al. (2013) | Muscle | Handgrip strength | Synbiotic | FOS + *Lactobacillus paracasei, L. rhamnosus, L. acidophilus*, and *Bifidobacterium lactis* | 10^8^-10^9^ CFU plus FOS | 10^8^-<10^10^ CFU/day* | Powder/sachets; maltodextrin placebo | 12 weeks |
| Qaisar et al. (2024) | Muscle | Handgrip strength | Mixed multistrain probiotic | Vivomixx 112: *Bifidobacterium spp., Streptococcus thermophilus, Lactobacillus*-related spp. | 112 x 10^9^ CFU/day | 10^10^-<10^12^ CFU/day | Capsules; inert placebo | 16 weeks |

**Notes.** Dose categories: 10^6^-<10^8^ CFU/day; 10^8^-<10^10^ CFU/day; 10^10^-<10^12^ CFU/day; >=10^12^ CFU/day; or not reported/not convertible when total daily CFU could not be derived from the published information. The category "separate single-strain probiotic arms" was used for one trial with distinct Lactobacillus-only and Bifidobacterium-only active arms. The term synbiotic was assigned to formulations containing live microorganisms plus a prebiotic substrate such as inulin, fructooligosaccharides, or fiber. Asterisked dose categories indicate that the available report did not always permit exact conversion to total daily CFU. The “Meta-analysis contribution” column indicates participation in an outcome-specific quantitative synthesis. Studies classified as “Qualitative only” remained included in the systematic review but did not contribute to a pooled effect estimate.

**Supplementary Table S3. Classification of included studies by strain type and reported dose range.** This table classifies the studies included in the qualitative synthesis according to intervention type. Dose ranges refer to reported daily CFU when available; some studies reported dose as product weight, concentration, or per-capsule CFU only and therefore could not be converted reliably to total daily CFU.

| **Instrument(s) reported in included studies** | **Included studies** | **Clinical populations in this review** | **Score range / score structure** | **Main domains assessed** | **Interpretative considerations for sensitivity, ceiling/floor effects, and pooling** | **Refs** |
| --- | --- | --- | --- | --- | --- | --- |
| MMSE and variants (MMSE, MMSE-J, MMSE-K, modified MMSE) | Akbari 2016; Akhgarjand 2022; Asaoka 2022; Azuma 2023 (baseline only); Chung 2014 (baseline only); Fei 2023; Kim 2021 (baseline only); Kobayashi 2019; Louzada 2020; Ruiz-Gonzalez 2025; Wang 2021; Xiao 2020 (baseline only) | Alzheimer's disease, mild cognitive impairment (MCI), suspected MCI, subjective memory complaints, healthy/community-dwelling aging adults, postoperative older adults | Brief global cognitive screening scale. Standard MMSE is typically scored from 0 to 30, with higher scores indicating better global cognition; country/language versions follow similar interpretive logic. | Orientation, registration, attention/calculation, recall, language, and visuospatial/constructional praxis. | Useful for global cognition and for moderate impairment, but may show ceiling effects in cognitively healthy or mildly impaired adults and floor effects in more advanced dementia. Educational and language effects should be considered. | S4-1 |
| MoCA and MoCA-J | Azuma 2023 (baseline only); Eastwood 2025 (baseline only); Fei 2023; Shi 2023 | MCI, healthy aging adults, suspected MCI or screening contexts | Brief multidomain screening tool, commonly scored from 0 to 30, with higher scores indicating better cognition. | Executive/visuospatial function, naming, attention, language, abstraction, delayed recall, and orientation. | Designed to be more sensitive than MMSE for mild impairment, but performance depends on education, language, cultural adaptation, and ceiling effects may occur in high-functioning participants. | S4-2 |
| RBANS | Kobayashi 2019; Shi 2023; Xiao 2020 | Subjective memory complaints, MCI/suspected MCI, healthy aging adults | Neuropsychological battery with total/index scores and domain scores; higher scores indicate better performance. Scoring depends on version and normative approach. | Immediate memory, visuospatial/constructional ability, language, attention, and delayed memory. | More domain-specific than MMSE and potentially informative for memory-related changes, but may be sensitive to baseline population differences, version/norms, and practice effects. In this review, RBANS studies were few and clinically heterogeneous. | S4-3 |
| ADAS-cog variants (ADAS-Jcog, ADAS-cog-13) | Asaoka 2022; Baek 2025 | MCI/suspected MCI | Alzheimer's disease cognitive assessment scale; higher scores generally indicate worse cognitive performance. Total range varies by version (e.g., 11-item versus 13-item forms and language adaptations). | Memory, language, praxis, orientation, and other cognitive functions relevant to AD-spectrum impairment. | Often used in AD/MCI trials and may be more sensitive to disease-related cognitive change than brief screening tools, but version differences and population severity affect interpretability. | S4-4 |
| CERAD-K | Kim 2021 | MCI in community-dwelling older adults | Korean adaptation of the CERAD neuropsychological assessment battery; includes several subtests rather than a single universal total-score range. | Verbal fluency, naming, verbal learning and memory, constructional praxis, recall/recognition, and related domains depending on the battery version. | Provides broader domain coverage than a brief global screener, but cross-study pooling is difficult because studies may report different subtests or composite outcomes. | S4-5, S4-6 |
| Cognitrax | Azuma 2023 | Community-dwelling adults with MCI but no dementia | Computerized cognitive battery corresponding to the Japanese version of CNS Vital Signs; reports domain-specific scores rather than one universal raw score range. | Composite domains may include memory, psychomotor speed, reaction time, attention, cognitive flexibility, processing speed, and executive function depending on the selected battery. | Can capture multiple domains and reaction-time based measures, but comparability depends on the selected modules and scoring algorithm. | S4-7 |
| NIH Toolbox Cognition Battery | Sanborn 2020 | Middle-aged and older adults | Computerized battery with standardized/normed scores; exact scoring depends on selected subtests and age-adjusted norms. | Executive function, attention, episodic memory, language, processing speed, and working memory depending on battery selection. | Useful for multidomain assessment and standardized comparisons, but pooling with MMSE/RBANS is not appropriate because construct and scale properties differ substantially. | S4-8 |
| Verbal learning and memory tests (RAVLT, VLT) | Bartos 2023; Chung 2014; Eastwood 2025; Hwang 2019 | Healthy older adults, MCI, mixed normal cognition/MCI groups | Typically scored using number of words recalled, learning across trials, delayed recall, or recognition; higher scores usually indicate better performance. | Verbal learning, immediate recall, delayed recall, recognition memory, and learning efficiency. | More domain-specific than global screening scales. Results can be influenced by language, education, list version, practice effects, and baseline memory status. | S4-9 |
| Attention and working-memory span tasks (DST, Digit task, RVIP, ACPT) | Baek 2025; Chung 2014; Hwang 2019; Ruiz-Gonzalez 2025 | MCI, healthy aging adults | Score structure varies by task: span length, accuracy, omission/commission errors, and reaction time. For DST, higher span generally indicates better attention/working memory. | Attention, vigilance, sustained attention, working memory, and processing speed. | Sensitive to fatigue, test modality, sensory/motor speed, and practice effects. Direct pooling across these tasks would require strong assumptions of construct equivalence. | S4-10, S4-11 |
| Trail Making Test (TMT) | Baek 2025; Bartos 2023; Lazou-Ahren 2025; Ruiz-Gonzalez 2025 | MCI, healthy aging adults, adults with systemic low-grade inflammation, mixed normal cognition/MCI groups | Usually scored by time to completion; lower time indicates better performance. Part A emphasizes processing speed; Part B adds set-shifting/executive demand. | Visual scanning, psychomotor speed, attention, cognitive flexibility, and executive function. | Performance is affected by motor speed, visual ability, education, and task familiarity. It is not directly comparable with global cognitive scales without standardization. | S4-12 |
| Stroop test | Baek 2025; Ruiz-Gonzalez 2025 | MCI and healthy aging adults | Scoring may use time, accuracy, errors, or interference scores; direction of effect depends on the metric reported. | Selective attention, response inhibition, cognitive control, and processing speed. | Sensitive to executive dysfunction but depends strongly on task version, color vision/reading ability, language, and scoring metric. | S4-13 |
| Clock Drawing Test (CDT) | Bartos 2023 | Community-dwelling adults with normal cognition or MCI | Multiple scoring systems exist; higher or lower scores may indicate better performance depending on the system used. | Visuospatial construction, executive planning, semantic knowledge, and praxis. | Brief and clinically useful, but score interpretation depends on the scoring system and may show ceiling effects in high-functioning adults. | S4-14 |
| Categorical Verbal Fluency Test (CFT) | Akhgarjand 2022 | Mild or moderate Alzheimer's disease | Usually scored as the number of correct category words generated within a fixed time; higher scores indicate better performance. | Semantic fluency, language retrieval, executive search strategy, and processing speed. | Sensitive to semantic memory and executive function, but strongly influenced by language, education, and category selection. | S4-15 |
| WAIS-III subtests | Bartos 2023 | Community-dwelling adults with normal cognition or MCI | Standardized subtest scores; exact score range depends on the specific WAIS-III subtest used. | Depending on subtest: working memory, processing speed, verbal comprehension, perceptual organization, and related domains. | Useful for domain-level assessment, but pooling requires knowledge of the specific subtest and scoring metric. | S4-16 |
| Corsi Block-Tapping Test / Corsi task / CBTT | Eastwood 2025; Ruiz-Gonzalez 2025 | Healthy aging adults | Typically scored as span length or number of correctly reproduced sequences; higher scores indicate better visuospatial short-term/working memory. | Visuospatial short-term memory and working memory. | Sensitive to visuospatial memory, but version, computerized versus physical administration, and practice effects influence comparability. | S4-17 |
| Go/no-go task | Eastwood 2025; Ruiz-Gonzalez 2025 | Healthy aging adults | Scored using accuracy, commission errors, omission errors, and/or reaction time; score direction depends on metric. | Response inhibition, sustained attention, and executive control. | Task parameters strongly influence sensitivity; reaction-time based metrics may be affected by motor speed and computer familiarity. | S4-18 |
| Tower of London | Ruiz-Gonzalez 2025 | Healthy adults over 55 years | Scoring may use number of moves, planning time, execution time, errors, or total achievement score depending on version. | Planning, problem solving, executive function, and working memory. | Sensitive to executive planning, but comparability depends on version, scoring, and motor/computer task demands. | S4-19 |
| Wisconsin Card Sorting Test | Ruiz-Gonzalez 2025 | Healthy adults over 55 years | Scoring commonly includes categories achieved, total errors, and perseverative errors; score direction depends on metric. | Cognitive flexibility, set shifting, abstract reasoning, and executive function. | Sensitive to executive dysfunction but influenced by learning, feedback processing, and task version; not directly comparable with global screening instruments. | S4-20 |
| Iowa Gambling Task | Ruiz-Gonzalez 2025 | Healthy adults over 55 years | Typically scored as net advantageous minus disadvantageous choices or block-wise performance; higher net scores generally indicate better advantageous decision-making. | Decision-making under uncertainty, reward/punishment learning, and affective executive function. | Interpretation depends on task version, learning trajectory, and motivational factors; not a general cognitive screening tool. | S4-21 |
| PEBL computerized task battery | Ruiz-Gonzalez 2025 | Healthy adults over 55 years | Open-source platform used to implement several cognitive tasks; scoring depends on the task implemented. | Varies by task: attention, memory, executive function, inhibition, decision-making, and processing speed. | PEBL is an administration platform rather than a single cognitive construct. Results should be interpreted at the task level rather than pooled as one instrument. | S4-22 |
| Japanese MCI Screen (JMCIS) | Xiao 2020 | Adults aged 50-79 years with suspected MCI | Brief memory-oriented screening approach derived from MCI Screen methodology; output depends on scoring algorithm/version. | Memory, delayed recall, cued recall, and screening-level cognitive classification. | Designed for early detection/screening contexts. Algorithmic scoring and language/cultural adaptation limit direct comparability with MMSE or RBANS. | S4-23 |
| Study-specific or locally reported cognitive tasks (ALBA, ABACO, PICNIR) | Bartos 2023 | Community-dwelling adults with normal cognition or MCI | Score structure was reported within the included study; no single internationally standardized score range was identified for these abbreviations. | Memory, naming, immediate recall, and/or broader cognitive performance as described in the included study. | These measures provide additional domain-level information but were not suitable for pooling because scoring, validation context, and comparability with other instruments were limited. | S4-24 |
| Functional questionnaires reported alongside cognitive outcomes (ADL, IADL, FAQ-CZ) | Akhgarjand 2022; Bartos 2023 | Alzheimer's disease; mixed normal cognition/MCI community-dwelling adults | Questionnaire-based functional scales; score direction and range vary by instrument/version. | Functional independence, instrumental daily activities, and everyday functional impact of cognitive impairment. | These are not cognitive performance tests and were not pooled as cognitive outcomes, but they provide clinical context for functional relevance. | S4-25, S4-26,  S4-27 |

**Notes.** When instruments have multiple versions or scoring systems, the table reports the general scoring structure rather than a single universal range. Higher scores indicate better performance unless otherwise stated. Instruments such as ADL, IADL, and FAQ-CZ are included because they were reported alongside cognitive outcomes in the included studies, but they were not treated as cognitive performance tests in the meta-analysis. Inclusion of a study in this table indicates that the instrument was reported; it does not necessarily indicate inclusion in the corresponding meta-analysis. Six unique studies were included in the pooled MMSE analyses. Ruiz-Gonzalez et al. and Wang et al. were retained in the qualitative synthesis but were not included in the pooled MMSE analysis.

**Supplementary Table S4. Cognitive instruments, scale properties, and clinical context across included studies.** This table summarizes only the cognitive instruments and related functional questionnaires reported in the included studies. It is intended to provide contextual information on scale structure, domain coverage, and interpretative considerations across clinical populations; it does not provide instrument-specific diagnostic sensitivity or specificity estimates, which vary by language, cut-off, age, education, and clinical setting.

References for Supplementary Table S4

S4-1. Arevalo-Rodriguez I, Smailagic N, Roqué-Figuls M, Ciapponi A, Sanchez-Perez E, Giannakou A, et al. Mini-Mental State Examination (MMSE) for the early detection of dementia in people with mild cognitive impairment (MCI). Cochrane Database Syst Rev. 2021;7(7). doi:10.1002/14651858.CD010783.pub3.

S4-2. Nasreddine ZS, Phillips NA, Bédirian V, Charbonneau S, Whitehead V, Collin I, et al. The Montreal Cognitive Assessment, MoCA: a brief screening tool for mild cognitive impairment. J Am Geriatr Soc. 2005;53(4):695–699. doi:10.1111/j.1532-5415.2005.53221.x.

S4-3. Duff K, Hobson VL, Beglinger LJ, O’Bryant SE. Diagnostic accuracy of the RBANS in mild cognitive impairment: limitations on assessing milder impairments. Arch Clin Neuropsychol. 2010;25(5):429–441. doi:10.1093/arclin/acq045.

S4-4. Kueper JK, Speechley M, Montero-Odasso M. The Alzheimer’s Disease Assessment Scale–Cognitive Subscale (ADAS-Cog): modifications and responsiveness in pre-dementia populations. A narrative review. J Alzheimers Dis. 2018;63(2):423–444. doi:10.3233/JAD-170991.

S4-5. Lee JH, Lee KU, Lee DY, Kim KW, Jhoo JH, Kim JH, et al. Development of the Korean version of the Consortium to Establish a Registry for Alzheimer’s Disease Assessment Packet (CERAD-K): clinical and neuropsychological assessment batteries. J Gerontol B Psychol Sci Soc Sci. 2002;57(1):P47–P53. doi:10.1093/geronb/57.1.P47.

S4-6. Lee DY, Lee KU, Lee JH, Kim KW, Jhoo JH, Youn JC, et al. A normative study of the CERAD neuropsychological assessment battery in the Korean elderly. J Int Neuropsychol Soc. 2004;10(1):72–81. doi:10.1017/S1355617704101094.

S4-7. Gualtieri CT, Johnson LG. Reliability and validity of a computerized neurocognitive test battery, CNS Vital Signs. Arch Clin Neuropsychol. 2006;21(7):623–643. doi:10.1016/j.acn.2006.05.007.

S4-8. Weintraub S, Dikmen SS, Heaton RK, Tulsky DS, Zelazo PD, Bauer PJ, et al. Cognition assessment using the NIH Toolbox. Neurology. 2013;80(11 Suppl 3):S54–S64. doi:10.1212/WNL.0b013e3182872ded.

S4-9. Schmidt M. Rey Auditory Verbal Learning Test: RAVLT: A Handbook. Los Angeles, CA: Western Psychological Services; 1996. 137 p.

S4-10. Woods DL, Kishiyama MM, Yund EW, Herron TJ, Edwards B, Poliva O, et al. Improving digit span assessment of short-term verbal memory. J Clin Exp Neuropsychol. 2011;33(1):101–111. doi:10.1080/13803395.2010.493149.

S4-11. Simon AJ, Gallen CL, Ziegler DA, Mishra J, Marco EJ, Anguera JA, Gazzaley A. Quantifying attention span across the lifespan. Front Cognit. 2023;2:1207428. doi:10.3389/fcogn.2023.1207428.

S4-12. Linari I, Juantorena G, Ibáñez A, Petroni A, Kamienkowski JE. Unveiling Trail Making Test: visual and manual trajectories indexing multiple executive processes. Behav Res Methods. 2022;54(2):927–940. doi:10.3758/s13428-021-01662-3.

S4-13. Scarpina F, Tagini S. The Stroop Color and Word Test. Front Psychol. 2017;8:557. doi:10.3389/fpsyg.2017.00557.

S4-14. Pinto E, Peters R. Literature review of the Clock Drawing Test as a tool for cognitive screening. Dement Geriatr Cogn Disord. 2009;27(3):201–213. doi:10.1159/000203344.

S4-15. Henry JD, Crawford JR, Phillips LH. Verbal fluency performance in dementia of the Alzheimer’s type: a meta-analysis. Neuropsychologia. 2004;42(9):1212–1222. doi:10.1016/j.neuropsychologia.2004.02.001.

S4-16. Wechsler D. WAIS-III: Wechsler Adult Intelligence Scale. 3rd ed. San Antonio, TX: Psychological Corporation; 1997.

S4-17. Kessels RPC, van Zandvoort MJE, Postma A, Kappelle LJ, de Haan EHF. The Corsi Block-Tapping Task: standardization and normative data. Appl Neuropsychol. 2000;7(4):252–258. doi:10.1207/S15324826AN0704_8.

S4-18. Criaud M, Boulinguez P. Have we been asking the right questions when assessing response inhibition in go/no-go tasks with fMRI? A meta-analysis and critical review. Neurosci Biobehav Rev. 2013;37(1):11–23. doi:10.1016/j.neubiorev.2012.11.003.

S4-19. Phillips LH, Wynn V, McPherson S, Gilhooly KJ. Mental planning and the Tower of London task. Q J Exp Psychol A. 2001;54(2):579–597. doi:10.1080/713755977.

S4-20. Nyhus E, Barceló F. The Wisconsin Card Sorting Test and the cognitive assessment of prefrontal executive functions: a critical update. Brain Cogn. 2009;71(3):437–451. doi:10.1016/j.bandc.2009.03.005.

S4-21. Bechara A, Damasio AR, Damasio H, Anderson SW. Insensitivity to future consequences following damage to human prefrontal cortex. Cognition. 1994;50(1–3):7–15. doi:10.1016/0010-0277(94)90018-3.

S4-22. Mueller ST, Piper BJ. The Psychology Experiment Building Language (PEBL) and PEBL Test Battery. J Neurosci Methods. 2014;222:250–259. doi:10.1016/j.jneumeth.2013.10.024.

S4-23. Trenkle DL, Shankle WR, Azen SP. Detecting cognitive impairment in primary care: performance assessment of three screening instruments. J Alzheimers Dis. 2007;11(3):323–335. doi: 10.3233/jad-2007-11309.

S4-24. Bartos A, Weinerova J, Diondet S. Effects of human probiotics on memory and psychological and physical measures in community-dwelling older adults with normal and mildly impaired cognition: results of a bi-center, double-blind, randomized, and placebo-controlled clinical trial (CleverAge biota). Front Aging Neurosci. 2023;15:1163727. doi:10.3389/fnagi.2023.1163727.

S4-25. Pfeffer RI, Kurosaki TT, Harrah CH Jr, Chance JM, Filos S. Measurement of functional activities in older adults in the community. J Gerontol. 1982;37(3):323–329. doi:10.1093/geronj/37.3.323.

S4-26. Lawton MP, Brody EM. Assessment of older people: self-maintaining and instrumental activities of daily living. Gerontologist. 1969;9(3):179–186. doi:10.1093/geront/9.3_Part_1.179.

S4-27. Katz S, Ford AB, Moskowitz RW, Jackson BA, Jaffe MW. Studies of illness in the aged. The Index of ADL: a standardized measure of biological and psychosocial function. JAMA. 1963;185(12):914–919. doi:10.1001/jama.1963.03060120024016.

**Supplementary Table S5. Targeted post hoc study-level sensitivity analyses for MMSE and handgrip strength.** Akhgarjand et al. was excluded at the study level by removing both active intervention arms simultaneously. Qaisar et al. was excluded from the handgrip-strength analysis. The overall MMSE estimate includes all remaining studies across the Alzheimer’s disease and broader cognitive-impairment subgroups. Analyses retaining only two unique studies were interpreted solely as exploratory stability checks and not as confirmatory evidence. Abbreviations: CI, confidence interval; MMSE, Mini-Mental State Examination.

**Panel A. Trials with reported post-intervention microbiota data (n = 10)**

| **Study** | **Microbiota assessment** | **Post-intervention composition/diversity findings** | **Persistence or engraftment** | **Association with cognitive or muscle outcomes** |
| --- | --- | --- | --- | --- |
| Asaoka et al. (2022) [22] | Feces at baseline and week 24; 16S rRNA V3-V4 sequencing with taxonomic profiling and Bray-Curtis ordination. | No significant overall intervention-related shift in genus-level community composition was detected. Baseline microbial differences were observed between participants with lower versus higher MMSE scores. | Not assessed. | Baseline/cross-sectional only: lower MMSE status was associated with lower Actinobacteria/Bifidobacterium and higher Prevotella and selected Clostridiaceae, Ruminococcaceae, and Phascolarctobacterium. No microbial change-outcome association was reported. |
| Azuma et al. (2023) [23] | Feces at baseline and week 12; genus-specific qPCR for Bifidobacterium (targeted abundance, not community profiling). | Significant: fecal Bifidobacterium increased within the active group and was higher than placebo at week 12 (7.71 ± 0.56 vs 7.31 ± 0.90 log cells/g; p = 0.031). Community alpha/beta diversity was not assessed. | Not assessed; genus-level qPCR cannot establish strain engraftment. | No direct association between the bacterial change and cognitive outcomes was reported. |
| Baek et al. (2025) [24] | Feces at baseline and week 12; 16S rRNA V3-V4 sequencing with taxonomic profiling. | Significant increases versus placebo were reported for Lactobacillaceae (p = 0.0247), Bifidobacteriaceae (p = 0.0436), and Lactobacillus (p = 0.0247); Bifidobacterium showed a non-significant trend (p = 0.0554). Community alpha/beta diversity was not reported. | Not assessed. | No direct microbiota-cognition association was reported; microbiota and cognitive changes were described as concurrent findings. |
| Eastwood et al. (2025) [27] | Stool collected before and after each 8-week crossover period; 16S rRNA sequencing with alpha/beta diversity and taxonomic analyses. | Mixed: no significant changes in Shannon or Simpson diversity, richness, beta diversity, or order/family-level composition. Lactococcus relative abundance increased significantly after probiotic versus placebo and versus baseline. | Not assessed after treatment withdrawal; the crossover washout was not used for strain-resolved persistence testing. | No direct statistical association between microbial changes and cognitive outcomes was reported. |
| Fei et al. (2023) [28] | Feces at baseline and week 12; 16S rRNA V3-V4 sequencing, Chao/Shannon measures, unweighted UniFrac PCoA, and taxonomic profiling. | Mixed/significant: species diversity did not differ, but beta diversity differed between groups after intervention (PERMANOVA p = 0.045). Bacteroidetes and ten reported genera/groups were significantly higher after probiotics: Blautia, Lachnospiraceae NK4A136 group, Muribaculaceae, Haemophilus, Coprococcus, Ruminococcus, Anaerostipes, Erysipelotrichaceae UCG-003, Prevotellaceae NK3B31 group, and Pantoea. | Not assessed. | No direct microbiota-cognition association analysis was reported; a mechanistic relationship was inferred from concurrent findings. |
| Hwang et al. (2019) [29] | Feces at baseline and week 12; targeted qPCR for Lactobacillus, Bifidobacterium, and Clostridium (not community profiling). | Mixed: Lactobacillus increased significantly with DW2009 (p = 0.045); Bifidobacterium (p = 0.789) and Clostridium (p = 0.936) did not change significantly. | Not assessed; genus-level qPCR cannot establish persistence of the administered strain. | No direct association between bacterial abundance and cognitive outcomes was reported. The reported BDNF-cognition relationship did not test a microbial association. |
| Kim et al. (2021) [30] | Feces at weeks 0, 4, 8, and 12; longitudinal 16S rRNA V3-V4 sequencing with alpha diversity and taxonomic analyses. | Mixed/significant: no significant alpha-diversity, OTU-level, or phylum-level changes. At genus level, Eubacterium, Allisonella, Clostridiales, and Prevotellaceae decreased significantly at week 12 in the probiotic group. | Not assessed after treatment withdrawal. | No direct microbial-cognitive score association was reported. Eubacterium and Clostridiales were negatively correlated with serum BDNF in the probiotic group, an indirect biomarker association. |
| Sanborn et al. (2020) [36]; companion report: Aljumaah et al. (2022) [44] | The included 2020 report collected stool but did not present microbiome results. The companion report analyzed baseline and 3-month samples using 16S rRNA sequencing, WGS in a subset, and LGG strain-specific qPCR. | Mixed/significant: aggregate alpha diversity and Bray-Curtis clustering were not significantly different by treatment, whereas paired Bray-Curtis beta diversity changed (p = 0.018). Treatment-related taxa differed by cognitive status. LGG DNA was higher after supplementation than at baseline/placebo (adjusted p < 0.0001), and Lactobacillus was 11.5-fold more abundant than with placebo. | End-of-intervention fecal recovery/persistence of LGG was assessed by strain-specific qPCR. No post-withdrawal follow-up was performed; durable persistence or engraftment was therefore not established. | Significant/direct: in participants with MCI, decreases in Prevotella and Dehalobacterium after LGG were reported as correlated with improved cognitive score. Baseline taxa-cognitive status associations were also reported. |
| Shi et al. (2023) [37] | Feces at baseline and week 8; 16S rRNA V3-V4 sequencing with alpha/beta diversity and taxonomic analyses. | Mixed/significant: alpha and beta diversity did not change significantly. Several taxa changed significantly, including decreases in Solobacterium and Oribacterium within the active group; after intervention, Bifidobacterium was higher and several other genera/groups were lower than placebo. | Not assessed. | No direct association between microbial changes and cognitive outcomes was reported. |
| Wang et al. (2021) [38] | Feces one day before treatment and on postoperative days 5-7; 16S rRNA V3-V4 sequencing with phylum- and genus-level relative abundance. | Inconclusive/non-significant for the intervention contrast: no significant between-group difference at phylum level, and the reported greater genus-level perioperative change in the probiotic group was also non-significant (p > 0.05). Some within-group genus-level changes were present, but these did not establish a significant between-group microbiota effect. Diversity indices were not reported. | Not assessed after treatment withdrawal. | No direct association between microbiota changes and postoperative MMSE change was reported. |

**Panel B. Trials without reported post-intervention microbiota composition/diversity results (n = 14)**

| **Study** | **Information available** | **Classification for Comment 5** |
| --- | --- | --- |
| Akbari et al. (2016) [20] | No fecal microbiota analysis; the inability to measure fecal bacterial loads was stated as a limitation. | Not measured. |
| Akhgarjand et al. (2022) [21] | The authors explicitly stated that changes in gut microbiota were not assessed. | Not measured. |
| Bartos et al. (2023) [25] | Stool was collected and microbiome/biological analyses were planned for separate publication, but no post-intervention microbiome results were reported in the included article. | Samples collected; results unavailable/not reported. |
| Chung et al. (2014) [26] | Reported cognitive, psychological, BDNF, and blood-viscosity outcomes; no fecal microbiota composition/diversity analysis was reported. | Not measured. |
| Kobayashi et al. (2019) [31] | Reported cognitive outcomes without a fecal microbiota composition/diversity analysis. | Not measured. |
| Lazou-Ahren et al. (2025) [32] | Fecal calprotectin and zonulin were measured, but these are inflammatory/barrier biomarkers rather than microbiota composition or diversity. | Biomarkers only; not counted as microbiome composition/diversity. |
| Louzada/Ribeiro et al. (2020) [33] | Reported systemic inflammation, symptoms, and gut-function outcomes without microbiota composition/diversity profiling. | Not measured. |
| Mehmandoust et al. (2024) [34] | Reported cognitive, functional, and metabolic outcomes without fecal microbiota composition/diversity analysis. | Not measured. |
| Ruiz-Gonzalez et al. (2025) [35] | Stool consistency/frequency was assessed, but microbial composition/diversity was not; the authors recommended bacterial determination in future work. | Stool symptoms only; not measured. |
| Xiao et al. (2020) [39] | No fecal microbiota comparison was performed; this was identified as a future research need. | Not measured. |
| Lee et al. (2021), TWK10 [40] | No fecal microbiota analysis; inability to collect fecal samples was identified as a limitation. | Not measured. |
| Lee et al. (2025), PS23 [41] | No fecal microbiota composition/diversity analysis was reported; future microbiota studies were recommended. | Not measured. |
| Neto et al. (2013) [42] | Reported body-composition and inflammatory outcomes without fecal microbiota composition/diversity analysis. | Not measured. |
| Qaisar et al. (2024) [43] | Plasma zonulin and systemic bacterial load were measured, but fecal microbiota composition/diversity was not. | Non-compositional biomarkers only; not counted. |

**Supplementary Table S6. Post-intervention gut microbiota outcomes, persistence or engraftment, and associations with cognitive or muscle outcomes across the 24 included trials.** At trial level, 10 of 24 studies provided post-intervention gut microbiota composition, diversity, or targeted bacterial-abundance data (eight broad community-profiling studies and two targeted-qPCR studies). Eight reported at least one statistically significant microbiota finding; Asaoka et al. found no clear intervention-related community shift, and Wang et al. yielded an inconclusive/non-significant intervention contrast. One trial assessed end-of-intervention fecal recovery of the administered LGG strain, but no study assessed durable persistence after treatment withdrawal. Two trials reported microbiota-cognition relationships, although only one linked intervention-related microbial changes to improved cognition. No study reported a microbiota-muscle outcome association.

**Notes.** Counts are trial-level. Aljumaah et al. (2022) is a companion microbiome report from the same randomized trial as Sanborn et al. (2020) and is not counted as an additional study. Targeted qPCR of selected bacterial groups was distinguished from broad community profiling. End-of-treatment detection of an administered organism was not interpreted as durable engraftment unless a post-withdrawal assessment was performed. Fecal inflammatory/barrier biomarkers, stool symptoms, and systemic bacterial load were not counted as microbiota composition or diversity assessments.

**Companion report.** Aljumaah MR, Bhatia U, Roach J, et al. The gut microbiome, mild cognitive impairment, and probiotics: a randomized clinical trial in middle-aged and older adults. Clinical Nutrition. 2022;41:2565-2576. doi:10.1016/j.clnu.2022.09.012.

**Abbreviations.** BDNF, brain-derived neurotrophic factor; LGG, Lactobacillus rhamnosus GG; MCI, mild cognitive impairment; MMSE, Mini-Mental State Examination; PCoA, principal coordinates analysis; qPCR, quantitative polymerase chain reaction; WGS, whole-genome shotgun sequencing.

**Supplementary Figure S1. Forest plots of MMSE domain-specific outcomes.** Random-effects meta-analyses of studies evaluating the effects of live microorganism-based interventions on orientation and recall components of the Mini-Mental State Examination (MMSE) in older adults. Each pooled analysis included three studies. Effect sizes are presented as Hedges’ g with 95% confidence intervals (95% CI), with positive values favoring the intervention group. No statistically significant pooled effects were observed for orientation (g = 0.11; 95% CI: −0.23 to 0.45; I² = 47.43%) or recall (g = 0.53; 95% CI: −0.80 to 1.86; I² = 96.19%). Language and visuospatial outcomes were synthesized narratively because only two studies reported comparable data for each domain and were therefore not pooled. Squares represent individual study effect sizes, with size proportional to study weight, and diamonds represent the pooled effect estimates.

**Supplementary Figure S2. Forest plots of RBANS domain-specific outcomes.** Random-effects meta-analyses of studies evaluating the effects of live microorganism-based interventions on domain-specific outcomes of the Repeatable Battery for the Assessment of Neuropsychological Status (RBANS) in older adults. Separate pooled analyses are shown for immediate memory, visuospatial/constructional abilities, language, attention, and delayed memory. Each RBANS domain-specific analysis included three unique studies and three intervention comparisons. Effect sizes are presented as Hedges’ g with 95% confidence intervals (95% CI). Positive effect sizes favor the intervention group over placebo. No statistically significant pooled effects were observed for immediate memory (g = 0.56; 95% CI: −0.19 to 1.31; I² = 89.27%), visuospatial/constructional abilities (g = 0.44; 95% CI: −0.24 to 1.13; I² = 87.34%), language (g = 0.14; 95% CI: −0.44 to 0.73; I² = 83.00%), attention (g = 0.03; 95% CI: −0.60 to 0.65; I² = 85.22%), or delayed memory (g = 0.41; 95% CI: −0.42 to 1.23; I² = 91.20%). Squares represent individual study effect sizes, with size proportional to study weight, and diamonds represent pooled effect estimates.

**Supplementary Figure S3. Targeted post hoc study-level sensitivity analysis of MMSE after excluding Akhgarjand et al**. Both intervention arms from this study were removed simultaneously. The two remaining studies in the Alzheimer’s disease subgroup yielded a pooled effect of g = 1.63 (95% CI: 1.21 to 2.06; I² = 0.00%). Across all remaining MMSE studies, the overall pooled effect was g = 0.94 (95% CI: 0.03 to 1.85; I² = 94.13%; p = 0.043). The test for subgroup differences was not statistically significant (Qb(1) = 2.74, p = 0.10). Because only two studies remained in the Alzheimer’s disease subgroup, this analysis should be interpreted solely as an exploratory stability check and not as confirmatory evidence.

**Supplementary Figure S4. Targeted post hoc study-level sensitivity analysis of handgrip strength after excluding Qaisar et al.** The analysis retained two unique studies and three intervention comparisons because Lee et al. contributed two active intervention arms; its shared placebo group was adjusted to prevent double counting. The pooled effect was attenuated and was no longer statistically significant (g = 0.23; 95% CI: −0.27 to 0.73; I² = 0.00%; p = 0.360). Because only two unique studies remained, this analysis should be interpreted solely as an exploratory stability check and not as confirmatory evidence.
